# Effect of the Nature of the Electrolyte on the Behavior of Supercapacitors Based on Transparent ZnMn_2_O_4_ Thin Films

**DOI:** 10.3390/nano13233017

**Published:** 2023-11-24

**Authors:** Juan José Peinado-Pérez, Maria Cruz López-Escalante, Francisco Martín

**Affiliations:** 1 Department of Applied Physics I, Faculty of Sciences, University of Málaga, Campus de Teatinos, E-29071 Málaga, Spain; q12pepej@uma.es; 2 Department of Chemical Engineering, Faculty of Sciences, University of Málaga, Campus de Teatinos, E-29071 Málaga, Spain; mclopez@uma.es

**Keywords:** energy storage, supercapacitor, ZnMn_2_O_4_, thin film, electrolyte, transparent

## Abstract

Transparent ZnMn_2_O_4_ thin films on indium tin oxide (ITO) were prepared through spray pyrolysis and implemented as electrodes in symmetric supercapacitors (SSCs). A specific capacitance value of 752 F g^−1^ at 0.5 A g^−1^ and a 70% retention over 3000 galvanostatic charge–discharge (GCD) cycles were reached with a 1.0 M Na_2_SO_4_ electrolyte in a three-electrode electrochemical cell. Analysis of the cycled electrodes with 1.0 M Na_2_SO_4_ revealed a local loss of electrode material; this loss increases when electrodes are used in SCCs. To avoid this drawback, solid polyvinylpyrrolidone-LiClO_4_ (PVP-LiClO_4_) and quasi-solid polyvinylpyrrolidone-ionic liquid (PVP-ionic liquid) electrolytes were tested in SSCs as substitutes for aqueous Na_2_SO_4_. An improvement in capacitance retention without a loss of electrode material was observed for the PVP-ionic liquid and PVP-LiClO_4_ electrolytes. With these non-aqueous electrolytes, the tetragonal structure of the ZnMn_2_O_4_ spinel was maintained throughout the cyclic voltammetry (CV) cycles, although changes occurred in the stoichiometry from ZnMn_2_O_4_ to Mn-rich Zn_1−x_Mn_3−x_O_4_. In the case of the electrolyte 1.0 M Na_2_SO_4_, the loss of Zn^2+^ led to the formation of MnO_2_ via Zn_1-x_M_3-x_O_4_. The location of the three SCCs in the Ragone plot shows supercapacitor behavior. The electrochemical results prove that the pseudocapacitance is the major contributor to the electrode capacitance, and the SCCs can therefore be considered as pseudocapacitors.

## 1. Introduction

There are currently several active approaches to promote the development of storage systems, as well as generation and storage together, to overcome the inherent seasonality of renewable energies. There are many devices that can store electric energy, such as rechargeable lithium-ion batteries, capacitors, electrochemical capacitors, and fuel cells. They all differ in their energy density, power density, efficiency, voltage window, and current density [1]. Electrochemical capacitors, also named supercapacitors (SCs), are devices with a promising future as energy storage systems because, compared to other systems, they offer a high power density, fast charge–discharge rate, excellent cycle stability, and low cost and they can be designed as eco-friendly systems with the proper selection of materials [2,3]. There is an emerging demand for transparent optoelectronic devices where transparent supercapacitors with high optical transmittance, but without sacrificing performance, will be needed.

The energy storage mechanism of SCs is due to the double-layer capacitance formed by the electrode–electrolyte interface and faradaic processes produced by reversible adsorption–desorption or oxidation–reduction chemical reactions (pseudocapacitance) [4]. SCs’ electrochemical performance is closely related to the electrode material, surface area, porosity, electrochemical activity, and kinetic characteristics of the electrodes [5]. Therefore, this implies that, in addition to being a suitable material selection, it is important to improve the ion and electron transport of the electrode, and the electrode/electrolyte interface [4,6,7].

Carbon-based materials, conducting polymers, and metal oxides are used as electrode materials. These materials have advantages and disadvantages. Carbon materials show great mechanical strength, excellent electronic conductivity, high specific surface area accessible to the electrolyte ions, and can be obtained from biomass [8,9]. Graphene oxide tends to aggregate and restack, making its surface less accessible to the electrolyte. Conducting polymers have a relatively high conductivity and capacitance compared to carbon-based electrode materials [10]. However, reduction–oxidation causes mechanical stress in conducting polymers, limiting their stability during charge–discharge cycles. Metal oxides, such as MnO_2_, exhibit high energy and power density. However, they suffer from poor electrical conductivity, capacitance fading, short cycling lifetime due to their intrinsic drawbacks of crystallographic instability, volume expansion, and severe aggregation during redox reactions [11]. Zhong-Shuai et al. [12] comparatively reported the pros and cons of graphene, metal oxides, and graphene/metal oxide composites.

Manganese oxide-based materials are interesting for SCs due to their high theoretical specific capacitance, excellent capacitive performance in aqueous electrolytes [13,14], multivalence, low cost, and environmental friendliness. Manganese can adopt ten oxidation states, 7+ through 3−, but only three oxidation states, 2+, 3+, and 4+, are usually observed [15,16]. Manganese forms numerous stable stoichiometric oxides (MnO_2_, Mn_2_O_3_, Mn_3_O_4_, and MnO) and metastable oxides (such as Mn_5_O_8_) in which Mn^2+^ and Mn^3+^ coexist [17]. Manganese oxides form a variety of polymorphs; for example, MnO_2_ may exist as crystalline α, β, γ,δ or ε and the oxyhydroxide MnOOH exhibits three natural polymorphisms $\left(\alpha ,\;\beta ,\;\gamma \right)$.

Manganese oxide MnO_2_ has a large theoretical specific capacitance of 1370 F g^−1^, but its high resistivity (10^−6^ S cm^−1^) reduces electronic conductivity substantially [18]. Electrodes with MnO_2_ as the principal active material suffer from a short cycle life, mainly due to Mn loss from the electrode through the disproportionation reaction to the production of soluble Mn^2+^ [2,19,20,21]. In order to avoid or reduce this unwanted behavior, some authors chose to limit the potential window to 0.1–1.0 V, reducing the probability of a MnO_2_ disproportion reaction [2], while other authors have tried to introduce different elements in the electrode matrix in order to increase its conductivity [19]. Reversible Zn^2+^ intercalation/deintercalation in the MnO_2_ host framework and the formation of ZnMn_2_O_4_ combined with H^+^ co-intercalation are recognized as the main energy storage mechanisms in Zn/MnO_2_ cells [22]. 

Aqueous Zn ion batteries adopt the controlled chemical extraction/insertion of Zn [23]. Zn forms, with Mn, various compounds ranging from mixed oxides to spinel compounds such as ZnMn_2_O_4_. ZnMn_2_O_4_ belongs to the AB_2_O_4_ spinel family, with Zn^2+^ and Mn^3+^ ions at the center of tetrahedral and octahedral sites, respectively, while the oxygen atoms are distributed over the corners of the octahedra and tetrahedra [24,25]. The Zn^2+^ in the spinel ZnMn_2_O_4_ can be replaced by divalent ions (Cd^2+,^ Ca^2+^, Mg^2+^, Mn^2+^), while Mn^3+^ can be replaced by trivalent ions (Al^3+^, Fe^3+^) [26]. However, the substitution of Zn^2+^ by Mn^2+^ would be compensated by the simultaneous oxidation of Mn^3+^ to Mn^4+^, which is described by the formula t-[Zn^2+^, Mn^2+^] o-[Mn^3+^, Mn^4+^, Zn^2+^]_2_ [O]_4_ (t, o, for tetrahedral and octahedral sites, respectively) [24]. Mn-rich spinels, such as Zn_x_Mn_3−x_O_4_ with x < 1, are stable at low temperatures [24]. Zn_x_Mn_3−x_O_4_ tetragonal spinel structures were found at room temperatures for a range of values of x from 0 to 1 [27]. With x > 1, Peitado et al. found a mixture of tetragonal and hexagonal spinel structures at high temperature [28]. ZnMn_2_O_4_ has been proposed as an anode for Li-ion batteries [29,30] and as electrodes for supercapacitors [31].

In this work, we analyzed the effect of an electrolyte on the spinel ZnMn_2_O_4_ when it is used as an electrode in symmetric SCs. Analysis of the cycled electrodes shows that with Na_2_SO_4_ aqueous electrolytes, irreversible zinc extraction and the local loss of electrode material occur. To avoid this drawback, quasi-solid PVP-ionic liquid and solid PVP-LiClO_4_ electrolytes were tested as substitutes for aqueous Na_2_SO_4_ in symmetric supercapacitors (SSCs). During CV cycles, the tetragonal spinel ZnMn_2_O_4_ undergoes a change in stoichiometry through the loss of Zn to Mn-rich phases, with Zn_1−x_Mn_3−x_O_4_ maintaining a tetragonal structure until it finally becomes MnO_2_, as is the case with the 1.0 M Na_2_SO_4_ electrolyte. This process is inhibited to a greater or lesser extent by the PVP-ionic liquid and PVP-LiClO_4_ electrolytes, resulting, after CV cycles, in a gradient of different stoichiometries in the composition of the thin film from Na_2_SO_4_ to Zn_0.67_Mn_2.33_O_4_.

## 2. Materials and Methods

### 2.1. Preparation of ZnMn_2_O_4_ Electrodes 

ZnMn_2_O_4_ electrodes were prepared through spray pyrolysis over commercial In_2_O_3_:Sn (ITO) glass (XOPGlass, Castellón, Spain). In this deposition method, the precursor solution was pushed through a syringe pump into the spray nozzle to be nebulized on the substrate with a stream of compressed air. The substrate was kept at a controlled temperature. The precursors zinc acetate (Zn(AC)_2_·2H_2_O) and manganese acetate (Mn(AC)_2_·4H_2_O) (Sigma-Aldrich, St. Louis, MO, USA) were dissolved in distilled water with concentrations of 0.005 M and 0.01 M, respectively. Regarding the experimental conditions of spray pyrolysis, the flow rate and substrate temperature were set at the values of 20 mL h^−1^ and 400 °C, respectively, while the deposition time varied from 2 to 15 min. The electrode size was 2.5 cm × 4.0 cm. 

### 2.2. Supercapacitor Assembly

**Figure 1:**
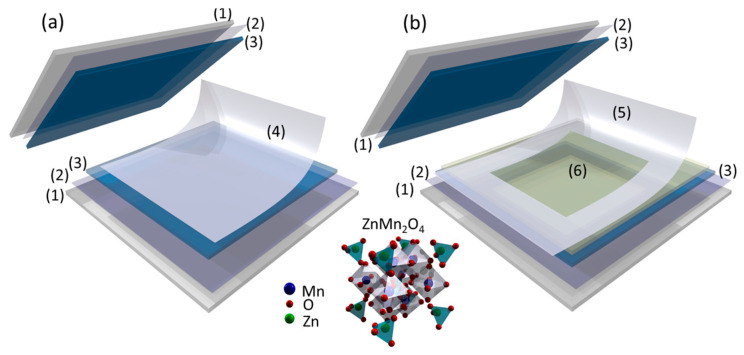
Scheme of the symmetric supercapacitors (**a**) using an acetate membrane soaked in 1.0 M Na_2_SO_4_ aqueous solution, and (**b**) with a Meltonix separation polymer and electrolyte formed by PVP- ionic liquid or PVP- LiClO_4_. (1) Glass, (2) ITO, (3) ZnMn_2_O_4_, (4) acetate membrane soaked with 1.0 M Na_2_SO_4_, (5) separation polymer (frame), (6) non-aqueous electrolyte (PVP-ionic liquid or PVP-LiClO_4_).

Three symmetric supercapacitors (SSCs) were assembled using two ZnMn_2_O_4_/ITO/glass electrodes and three different electrolytes (Figure 1). The first electrolyte was prepared using an acetate film soaked in 1.0 M Na_2_SO_4_ (Sigma-Aldrich, St. Louis, MO, USA) (Figure 1a); the second with 6.0 g of LiClO_4_ (Sigma-Aldrich, St. Louis, MO, USA) and 6.0 g of polyvinyl pyrrolidone (PVP) (Mw:1,300,000) (Sigma-Aldrich, St. Louis, MO, USA) dissolved in 75.0 mL of ethanol (Panreac Quimica, Barcelona, Spain) (named as PVP-LiClO_4_). This solution was screen-printed onto the ITO substrate and dried (Figure 1b). The ionic conductivity of the solid electrolyte obtained was 1.89 10^−4^ S cm^−1^; however, this conductivity may decrease due to the loss of residual ethanol from the electrolyte [32]. The third electrolyte was prepared with 9.0 g of commercial ionic liquid, 1-(2-hydroxyethyl)-3-methylimidazolium tetrafluoroborate [HEMIm][BF_4_] (Io-li-tec, Heilbronn, Germany), together with 16.0 g of PVP (named PVP-ionic liquid) dissolved in 55 mL of methanol. [HEMIm][BF_4_] ion gel exhibits an electrochemical stability window of, ca., 5.0 V and an ionic conductivity of 5.7 10^−3^ S cm^−1^ at room temperature [33]. A 25 µm Meltonix film (Solaronix, Aubonne, Switzerland) was used to prevent the direct contact between electrodes (Figure 1b). A Meltonix polymer was also used to seal the SSCs by heating it to 60 °C. The electrode available area in the SSC_S_ was 2 × 2 cm^2^. The PVP-ionic liquid and PVP-LiClO_4_ electrolytes had a pH of about 6, and the pH of the aqueous electrolyte 1.0 M Na_2_SO_4_ was 6.2.

### 2.3. Characterization Methods 

The thin-film crystalline structure was examined using an X-ray EMPYREAN diffractometer (PANanalytical, Malvern, UK). X-ray diffraction (XRD) spectra were recorded in the theta-theta transmission configuration, placing the sample between two Kapton foils, and by using a focusing mirror and the PIXcel 3D detector (working in 1D mode) with a step size of 0.013º (2θ). The diffractograms were recorded between 10° and 80° in 2θ with a total measuring time of 60 min. The morphology of the electrodes was studied using a field emission scanning electron microscope (FE-SEM), Helios Nanolab 650 dual beam instrument (Thermo Fisher Scientific, Waltham, MA, USA). High-resolution transmission electron microscopy (HRTEM) images and energy-dispersive X-ray spectroscopy (EDS) images were obtained on Talos F200X equipment (Thermo Fisher Scientific, Waltham, MA, USA). Optical transmittance measurements were carried out using a Varian Cary 5000 model spectrophotometer (Agilent, Santa Clara, CA, USA) with an integrating Spectralon sphere. X-ray photoelectron spectra (XPS) were recorded on a Physical Electronics PHI 5700 spectrometer (Physical Electronic, Chanhassen, MN, USA) using monochromatic Mg radiation. Adventitious C1s at 284.8 eV was used for charge shift correction.

### 2.4. Electrochemical Measurements

Electrochemical performance of ZnMn_2_O_4_/ITO electrode was measured in a typical three-electrode electrochemical cell with a saturated calomel electrode (SCE) as reference electrode and platinum as counter-electrode with 1.0 M Na_2_SO_4_ solution as electrolyte. The electrochemical properties were investigated through cyclic voltammetry (CV), galvanostatic charge–discharge (GCD), and electrochemical impedance spectroscopy (EIS). These electrochemical measurements were carried out on a Biologic VSP potentiostat (Biologic, Knoxville, TN, USA). Due to the gel-like nature of the PVP-ionic liquid and quasi-solid nature of the PVP-LiClO_4_ electrolytes, it was not possible to perform electrochemical tests in a three-electrode electrochemical cell. The electrochemical characterization of the SSCs was carried out with a two-electrode configuration for the three electrolytes tested. The specific capacitance *C* of the CVs and the GCD discharge curve (F·g^−1^), energy density *E* (Wh·kg^−1^), power density *P* (W·kg^−1^), and Coulombic efficiency η (%) were calculated according to Equations (1)–(5):

(1) $$C=\frac{\int i\left(u\right)du}{m\cdot v\cdot \Delta V}$$

(2) $$C=\frac{I\cdot \Delta t}{m\cdot \Delta V}$$

(3) $$E=\frac{0.5\cdot \;C\cdot \;\Delta V^{2}}{3.6}$$

(4) $$P=\frac{E}{\Delta t}\cdot 3600$$

(5) $$\eta =\frac{t_{d}}{t_{c}}\cdot 100$$

where $i\left(u\right)$ is the voltammetric current (A), m is the mass of active material (g), v is the potential scan rate (V·s^−1^), ΔV is the potential window of CV of the discharge curves (V), I is the applied current (A), Δt  and $t_{d}$ are the discharge times (s), and $t_{c}$ is the charge time (s). For SSCs where both electrodes have the same capacitance, the total capacitance is half of the electrode capacitance.

## 3. Results and Discussion

### 3.1. Characterization of the ZnMn_2_O_4_ Thin-Film Electrodes Obtained

#### 3.1.1. Chemical and Morphological Characterization

**Figure 2:**
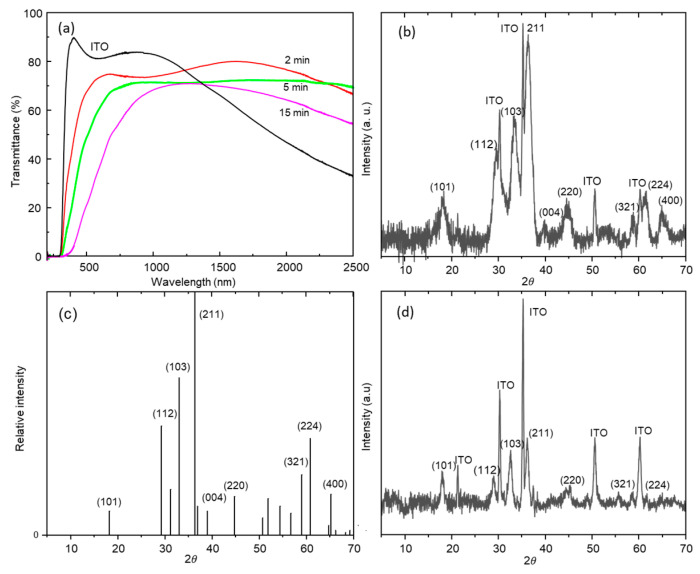
(**a**) Optical transmittance spectra of the ZnMn_2_O_4_/ITO/glass electrodes at different deposition times; (**b**) XRD pattern of the as-deposited electrode of ZnMn_2_O_4_ on ITO corresponding to deposition time of 15 min, (**c**) XRD standard diffraction pattern of ZnMn_2_O_4_ PDF 01-071-2499, (**d**) 5 min.

The ZnMn_2_O_4_/ITO/glass electrodes are transparent, and their light transmittance depends on the ZnMn_2_O_4_ film thickness (Figure 2a), which is a function of the deposition time (Figure 1a). Table S1 shows the optical parameters of the thin films. The importance of the electronic conductivity of the ZnMn_2_O_4_ electrode on the specific capacitance was indicated by Zhao et al. [29]. The sheet resistance of the ITO substrate, measured using the four-point probe technique (Ossila, Sheffield, UK), was 19.93 Ω per square, and that for the thin film of ZnMn_2_O_4_ was 447.97 Ω per square with a deposition time of 2 min. Thicker ZnMn_2_O_4_ films were more resistive and their specific capacitance decreased. For this reason, films with a deposition time of 2 min and a thickness of around 35 nm were selected to form the electrodes. The 2 min layer showed the best compromise between electrical conductivity, transparency, and specific capacitance.

Figure 2b–d show the grazing incidence X-ray diffraction spectra of the ZnMn_2_O_4_ films with deposition times of 5 and 15 min. The ZnMn_2_O_4_ films grown with a deposition time of 2 min did not show diffraction peaks. The identified XRD peaks (Figure 2b–d) correspond to the ITO substrate and tetragonal ZnMn_2_O_4_ hetaerolite, International Centre for Diffraction Data, Power Diffraction File (PDF) 01-071-2499 (Figure 2c). No peaks of ZnO, ZnMnO_3,_ or Mn oxides were identified by XRD. 

**Figure 3:**
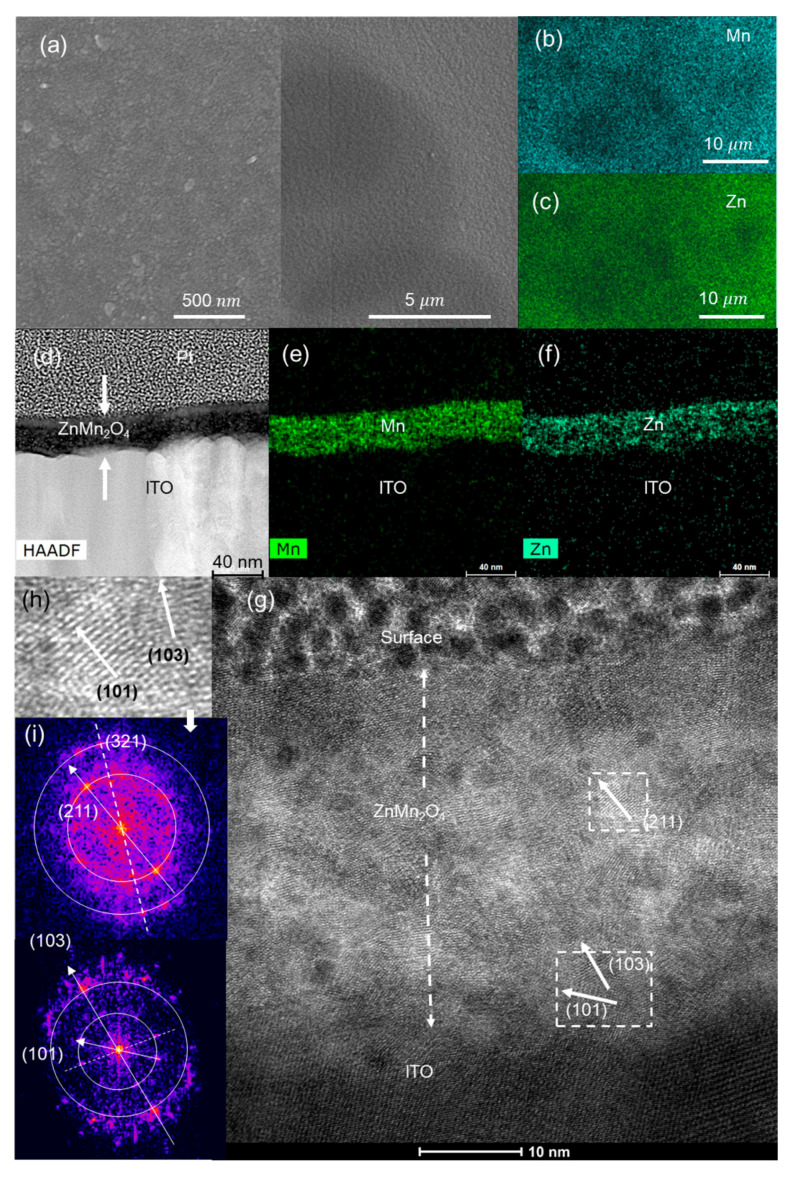
As-deposited ZnMn_2_O_4_ electrode: (**a**) SEM, (**b**) Mn, (**c**) Zn EDS images of the electrode surface; (**d**) HAADF; (**e**) Zn and (**f**) Mn EDS images of the electrode cross-section; (**g**) HRTEM image of the cross-section; (**h**) magnification of the marked zone; (**i**) FFTs of the film cross-section.

The SEM image of the ZnMn_2_O_4_ surface (Figure 3a) shows a superficial pattern of circles due to spray droplets. The chemical element surface maps using EDS (Figure 3b,c,e,f) indicate that Mn and Zn are homogeneously distributed on the surface and throughout the cross-section. Figure 3g shows an HRTEM image of the cross-section of the ZnMn_2_O_4_ thin film. Crystal planes corresponding to the most intense XRD peaks (103) (211) of the hetaerolite are indicated in the fast Fourier transform (FFT) (Figure 3i) and HRTEM images (Figure 3g,h). 

**Figure 4:**
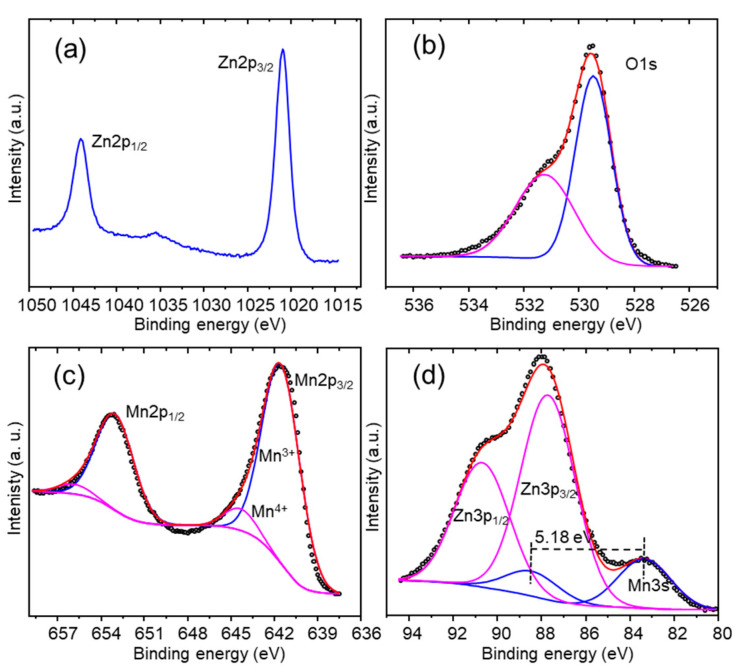
XPS spectra of (**a**) Zn2p, (**b**) O1s, (**c**) Mn2p, (**d**) Zn3p-Mn3s of the as-deposited ZnMn_2_O_4_ electrode.

XPS analysis of the surface was carried out by recording the O1s, Zn2p, Zn3p, Zn LMM, Mn2p, Mn3p, and Mn3s XPS regions. Figure 4a shows the two Zn^2+^ characteristic peaks corresponding to Zn2p_3/2_ and Zn2p_1/2_ at binding energies of 1021.24 and 1044.34 eV, respectively, and with a spin-splitting $\Delta E_{Zn2p}$ of 23.1 eV, in agreement with the literature [34,35,36]. O1s was deconvoluted in two peaks (Figure 4b), at 529.5 eV, corresponding to the (Zn/Mn)–O metal bond, and at 531.2 eV for OH groups adsorbed on the surface, following the proposal of other authors [26,37,38]. The width and the asymmetry of the Mn2p core level peaks (Figure 4c) indicate the presence of manganese in at least two different oxidation states; consequently, the Mn 2p_3/2_ main peak was deconvoluted in two components with a $\;\Delta E_{Mn2p}$ splitting of 11.5 eV [26,39]: the Mn2p_3/2_ main peak contribution at 641.4 eV of the Mn^3+^, and the component at 642.4 eV attributed to Mn^4+^ [40].

The presence of Mn^4+^ in the spinel ZnMn_2_O_4_ is coherent with the findings of other authors [37,39]. Mn3s shows two multiplet split components caused by the coupling of non-ionized 3s electrons with 3d valence band electrons (Figure 4d). However, in this case, the analysis of the Mn3s is substantially more complicated because the Zn3p signal is located in the same binding energy region as Mn3s, with Zn3p multiplet split components for Zn^2+^ with values of 87.7 eV and 90.7 eV for Zn3p_3/2_ and Zn3p_1/2_, respectively. The magnitude of peak splitting of Mn3s $\left(\Delta E_{Mn3s}\right)$ has been reported as 5.79 eV, 5.50 eV, 5.41 eV, and 4.79 eV for MnO, Mn_3_O_4_, Mn_2_O_3_, and MnO_2_, respectively [41,42]. To carry out the deconvolution of the Mn3s-Zn3p region (Figure 4d), $\Delta E_{Mn3s}$ (eV) was estimated using the equation of Beyreuther at al. (Equation (6)) [43].

(6) $$\upsilon_{Mn}=9.67-1.27\;\Delta E_{Mn3s}$$

where $\upsilon_{Mn}$ is the average oxidation state. According to the deconvolution of the Mn2p XPS signal (Figure 4c), the atomic ionic ratio Mn^3+^/Mn^4+^ is 10.63, meaning a value of $\upsilon_{Mn}$ = 3.09, and $\Delta E_{Mn3s}$= 5.18 eV (Equation (6)). This value of $\Delta E_{Mn3s}$= 5.18 eV was used for the deconvolution of the Mn3s signal (Mn3s-Zn3p), which is shown in Figure 4d. 

#### 3.1.2. Electrochemical Characterization

**Figure 5:**
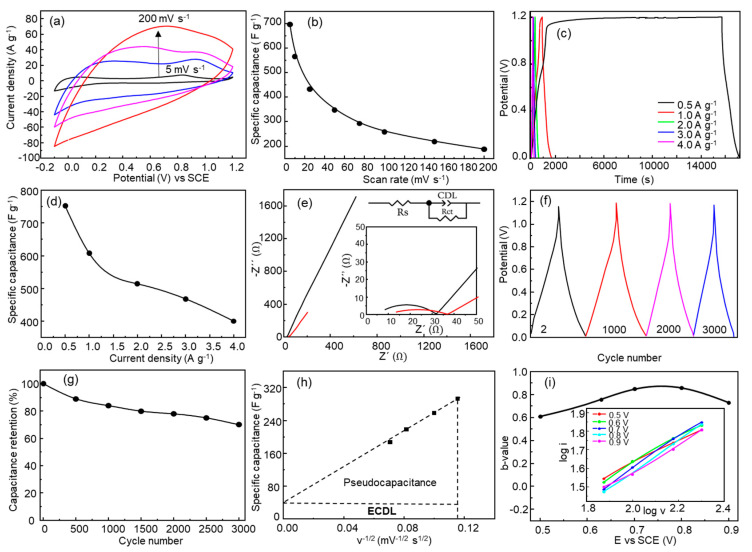
(**a**) Cyclic voltammetry curves of ZnMn_2_O_4_ electrode measured at different scan rates: 5, 50, 100, and 200 mV s^−1^; (**b**) specific capacitance calculated as a function of scan rate; (**c**) GCD curves at current densities of 0.5, 1.0, 2.0, 3.0, and 4.0 A g^−1^; (**d**) specific capacitance calculated as a function of current density; (**e**) Nyquist plot for ZnMn_2_O_4_ thin film (black: before; red: after cycling), inset: zoom of the high-frequency region (black: before; red: after CV cycles); (**f**) GCD for different number of cycles, (**g**) GCD capacitance retention; all the electrochemical analysis was carried out in 1.0 M Na_2_SO_4_ electrolyte; (**h**) specific capacitance vs. v^1/2^; (**i**) b parameter vs. the potential, inset: log i vs. log v, v scan rate (mV s^−1^).

A three-electrode electrochemical cell with 1.0 M Na_2_SO_4_ aqueous solution as the electrolyte was used to characterize the behavior of the ZnMn_2_O_4_/ITO working electrode. Pt was used as the counter-electrode and a saturated calomel electrode was used as a reference. Figure 5a and Figure S1 show the cyclic voltammetry curves (CV) ranging from −0.1 to 1.2 V at different scan rates from 5 mV s^−1^ to 200 mV s^−1^. The highest specific capacitance achieved (Equation (1)) was 697 F g^−1^ at 5 mV s^−1^, which is better than the other values reported for ZnMn_2_O_4_ obtained through spray pyrolysis; for instance, 530 F g^−1^ at 10 mV s^−1^ was reported by Boukmouche et al. [44], and 155 F g^−1^ at 2 mV s^−1^ was reported by Guo et al. [45]. Table S2 shows, comparatively, the results obtained by other authors. The specific capacitance undergoes a progressive decrease to 187 F g^−1^ at 200 mV s^−1^ due to the diffusion limitation related to a higher potential scan rate (Figure 5b) [46,47,48]. The square-like shape of the CV curves is due to the contribution of the electric double-layer capacitance and the pseudocapacitance. The faradaic contribution has been reported as a non-symmetric redox process related to the reversible extraction of Zn^2+^ ions from the ZnMn_2_O_4_ spinel according to reactions described in Equations (7) and (8) [20,49,50,51]. The anodic peak at 0.83 V (Figure 4a) contributes to the faradaic capacitance. The shift in the anodic peak from 0.83 V to 0.94 V would be due to polarization [52]. The reported equilibrium potential of the reaction (Equation (7)) is E_SHE_= 0.72 V (E_SCE_ = 0.961 V) [22]:

(7) $$ZnMn_{2}O_{4}\;\;↔Zn_{1-x}Mn_{3-x}O_{2}+x\;Zn^{2+}+2\;x\;e^{-}\;$$

which leads to the formation of $\;MnO_{2}$:

(8) $$ZnMn_{2}O_{4}↔\;\;2\;\;MnO_{2}+Zn^{2+}+2\;e^{-}\;$$

The reversible intercalation/deintercalation of Zn^2+^ in the MnO_2_ matrix with the formation of ZnMn_2_O_4_, shown in Equation (8), has been indicated as the main energy storage mechanism in Zn/MnO_2_ batteries [22,53].

Figure 5c shows the galvanostatic charge–discharge test (GCD) with applied current densities from 0.5 A g^−1^ to 4.0 A g^−1^. The GCD graphs present a quasi-symmetric triangular shape at higher specific current densities, 3.0 A g^−1^ and 4.0 A g^−1^, indicating the pseudocapacitive behavior of the ZnMn_2_O_4_ electrode due to the combination of the surface capacitive reactions and the redox reactions at the electrode–electrolyte interface, following Zn^2+^ extraction/insertion. Using Equation (2), specific capacitances from 752 F g^−1^ at 0.5 A g^−1^ to 400 F g^−1^ at 4.0 A g^−1^ were obtained (Figure 5d). The specific capacitance of 752 F g^−1^ (0.5 A g^−1^) is better than other specific capacitance values reported for electrodes based on manganese oxides obtained through spray pyrolysis, for example, with ternary compositions, such as M:Mn_3_O_4_, where M = Ce, Cr, Cu, and Ni were specifically reported as showing capacitances between 134 and 184 F g^−1^ at 0.5 A g^−1^, or 460 F g^−1^ at a scan rate of 5 mV s^−1^ for Ni:Mn_3_O_4_ [54,55,56,57]. 

Electrochemical impedance spectroscopy (EIS) measurements were performed in 1.0 M Na_2_SO_4_ electrolytes in the 10^−2^–10^5^ Hz frequency range. Figure 5e shows the Nyquist plots for the as-deposited ZnMn_2_O_4_ electrode and after 3000 CV cycles. The impedances were simulated using the equivalent circuit shown in the inset of Figure 5e. The elements of the equivalent circuit are the solution resistance (R_s_), the charge-transfer resistance (R_ct_), and the electrochemical double-layer capacitance (EDLC). The Nyquist curves consisted of a small semicircle at high frequency for the charge–transfer resistance (R_ct_) and a sloping line at the low-frequency region. The R_ct_ values were 25 Ω and 27 Ω for the electrode before and after CV cycles, respectively. Smaller R_ct_ is beneficial to the charge transfer. The R_s_ values obtained from the Nyquist plots were 7.0 Ω and 10.5 Ω for the non-cycled and cycled electrodes, respectively. Smaller values of R_s_ favor the diffusion of electrolyte ions [9,58].

Figure 5f shows the initial cycle and cycles with 1000, 2000, and 3000 galvanostatic charge–discharge at 2.0 A g^−1^ in a 1.0 M Na_2_SO_4_ electrolyte. As shown in Figure 5g, ZnMn_2_O_4_ electrodes exhibit a specific capacitance retention of 70% after 3000 GCD cycles.

The specific capacitance of the electrode is determined by the sum of the double-layer capacitance (EDLC) and the pseudocapacitance due to redox, intercalation, and diffusion processes within the active material [55,56,57]. The specific capacitance was plotted against the inverse of the square root of scan rate, v^−1/2^ (mV^−1//2^ s^1/2^), to determine the contribution of EDLC and pseudocapacitance (Figure 5h) through linear fitting. At a lower CV scan rate, the plot’s specific capacitance deviates from linearity and those values were excluded for the linear fitting (Figure 5h). The extrapolation of the linear fit to the y-axis assumes that the specific capacitance was expected to have an electrostatic origin [57], and the triangular area was assumed to indicate the pseudocapacitance. Therefore, from Figure 5h, it is deduced that the contribution to the specific capacitance is mainly from pseudocapacitive processes. The y-intercept of the linear fit determines the capacitance through EDLC as 24 F g^−1^ (3.4%), and the triangular area determines the capacitance provided by the pseudocapacitance as 673 F g^−1^ (96.6%), i.e., the electrode stores 96.6% of the charge based on Zn^2+^ intercalation or redox reactions and 3.4% of the charge based on the capacitive mechanism [55]. In addition, the current resulting from the voltammetric response at a specific voltage can be related to the scan rate according to the power-law formula (Equation (9)) [59]:

(9) $$i=a\;v^{b}$$

where *b* is 0.5 for battery-like behavior and 1 for typical EDLC behavior [60]. The representation of log *i* vs. log *v* is shown in the inset of Figure 5i, and the values of *b* are shown in Figure 5i. Figure 5i shows that at the smallest potential, *b* approaches 0.5, indicating that the diffusion-controlled processes, and battery-like behavior, predominate. However, as the potential increases, *b* becomes closer to 1, indicating an increase in capacitive contributions related to non-diffusive controlled processes such as EDLC [52,61,62,63].

### 3.2. Characterization of the ZnMn_2_O_4_ Thin-Film Electrodes after Cycling Process

#### Chemical and Morphological Characterization

**Figure 6:**
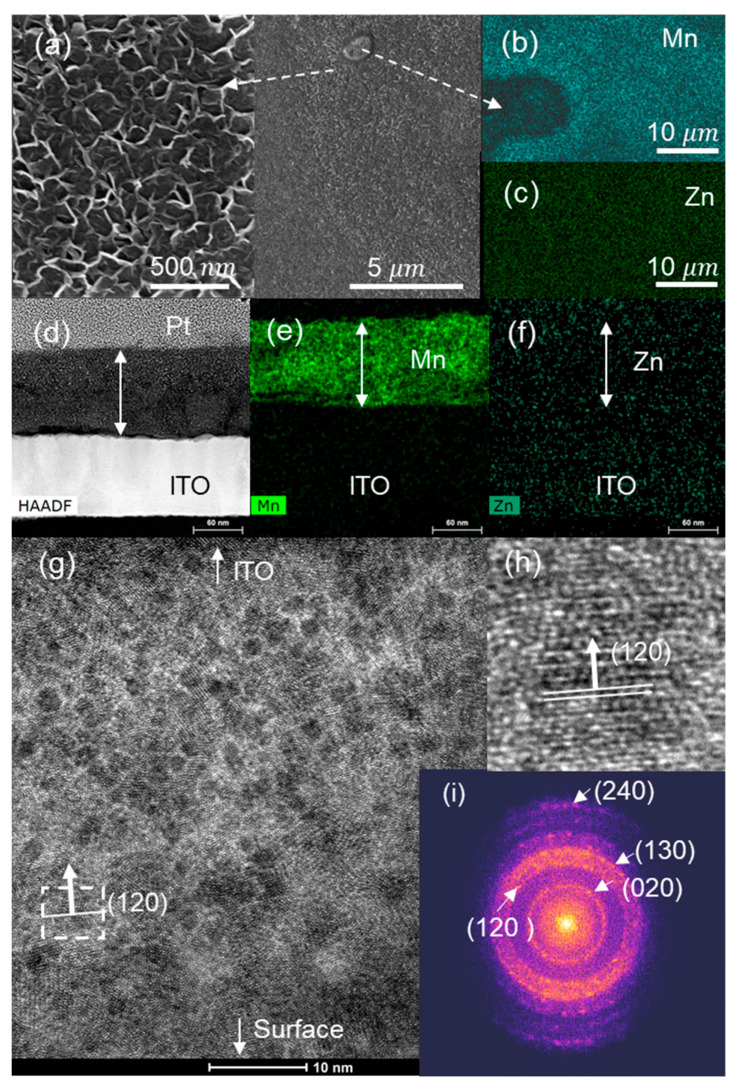
ZnMn_2_O_4_ electrode after 300 CV cycles: (**a**) SEM; (**b**) Mn and (**c**) Zn EDS images of the electrode surface; (**d**) HAADF; (**e**) Mn and (**f**) Zn EDS images of the electrode cross-section; (**g**) HRTEM image of the cross-section, (**h**) magnification of the marked zone; (**i**) FFTs of the film cross-section.

To eliminate traces of the precipitated electrolyte salt, the cycled electrodes were rinsed with water. Figure 6a shows the SEM image of the ZnMn_2_O_4_ electrode surface after 3000 CV cycles. The surface appears more textured and lacks the circular surface marks of spray droplets. This change in the surface texture is produced through chemical modification of the ZnMn_2_O_4_ according to Equations (7) and (8). The SEM-EDS images of the cycled electrode show holes corresponding to local loss of the electrode material (Figure 6a,b). The loss of Zn is also shown through EDS (Figure 6c), yielding a Mn/Zn average atomic ratio of 2 for the as-deposited electrode (Figure S2), and an average local value of 42 for the cycled electrode, with points where Zn was not detected (Figure S3). The HRTEM-EDS mapping images (Figure 6f) also show Zn loss in the cross-section of the film. The loss of electrode material is due to the solubilization of the Mn and Zn [18,49]. The Zn loss leads to irreversibility in the reactions of Equations (7) and (8), and, therefore, there is an evolution of the composition of the electrode with loss of zinc during cycling. 

**Figure 7:**
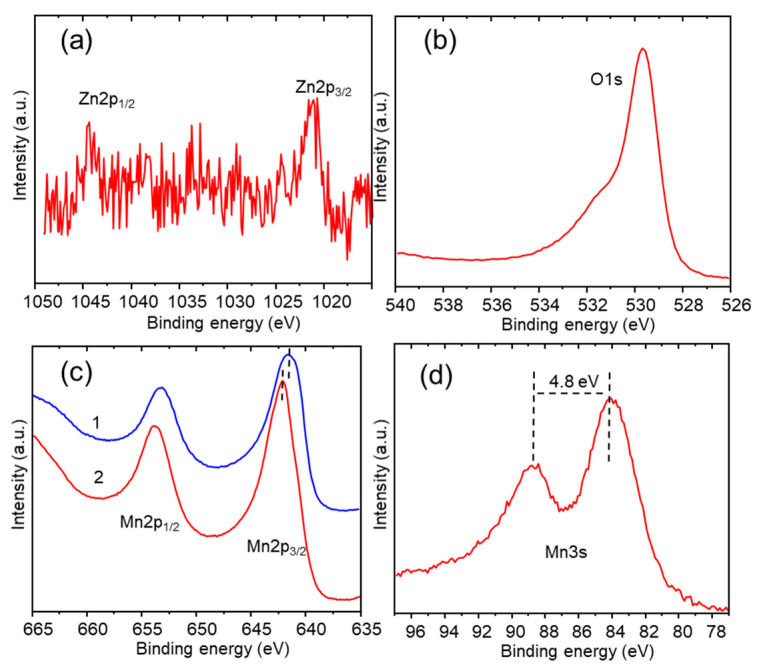
XPS spectra of (**a**) Zn2p, (**b**) O1s, (**c**) Mn2p, (**d**) Zn3p-Mn3s of the ZnMn_2_O_4_ electrode after 3000 CV cycles.

The XPS analysis (Figure 7a) of the electrode surface showed that after 3000 CV cycles, the intensity of the Zn2p_3/2_ peak at 1021.3 eV decreased remarkably. The shift in the Mn2p_3/2_ peak to a higher binding energy of 643.1eV (Figure 7c) is due to the increased contribution of the Mn^4+^ oxidation state. The Mn3s peak splitting ΔE_Mn3s_ = 4.8 eV for the cycled sample (Figure 7d), $\upsilon_{Mn}$= 3.57 according to Equation (8), leading to a MnO_1.7_ stoichiometry, indicates the transformation of ZnMn_2_O_4_ to MnO_2_ [41], in addition to the reversible transformation of ZnMn_2_O_4_ to MnO_2_. MnO_2_ may participate in pseudocapacitive reactions with the electrolyte (Equations (10) and (11)):

(10) $$\mathrm{Mn}O_{2}+H^{+}+e^{-}↔\;\mathrm{MnOOH}$$

(11) $$\mathrm{Mn}O_{2}+Na^{+}+e^{-}↔\;\mathrm{MnOONa}$$

The formation of MnOOH was related to a better capacitive performance since it facilitates ionic exchange [64,65,66]. The mild-acidic condition of the aqueous Na_2_SO_4_ electrolyte should prevent poor cycling performance due to the formation of species such as Mn(OH)_2_ or ZnO. However, it does not prevent dissolution of Zn^2+^. The loss of capacitance retention during GCD cycling (Figure 5g) is a consequence of the loss of Zn and the partial dissolution of Mn in the electrolyte [22,53]. Due to film thickness and low crystallinity, it was not possible to obtain grazing XRD spectra to identify the present compounds in the electrode. However, according to the HRTEM images of the cross-section (Figure 6g,h) and the corresponding FFT (Figure 6i), interplanar distances compatible with δ-MnO_2_ (PDF 04-005-4334) were obtained; these distances were also compatible with the Mn-rich spinel Zn_1−x_ Mn_2–y_O_4_, with values of x and y between the values of Zn_0.244_Mn_2.758_O_4_ (PDF 01-070-9109) and Zn_0,02_Mn_2.98_O_4_ (PDF 04-012-4910). These findings are supported by the values of the Mn/Zn ratio found through EDS (Figure S3). 

### 3.3. Symmetrical Supercapacitor

#### 3.3.1. 1.0 M Na_2_SO_4_ as Electrolyte

**Figure 8:**
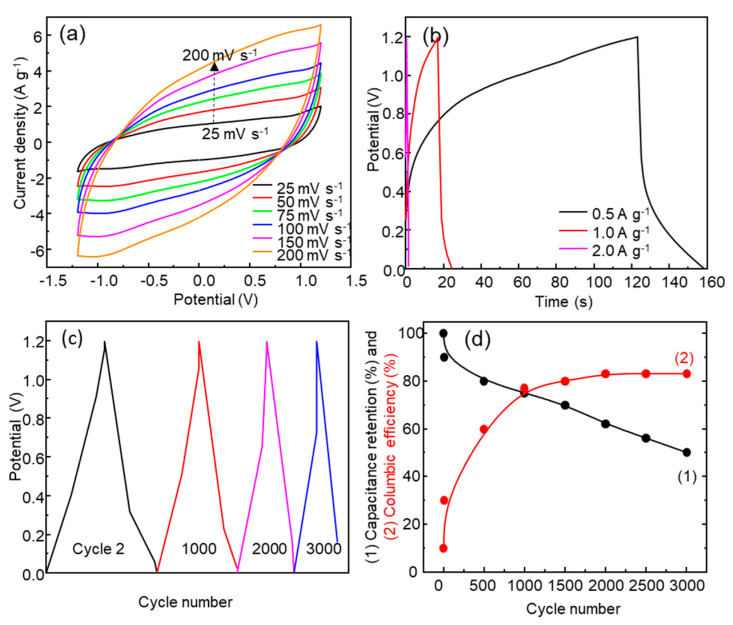
(**a**) Cyclic voltammetry curves with ±1.2 V potential window at scan rates from 25 to 200 mV s^−1^; (**b**) GCD at different current densities of 0.5 A g^−1^, 1.0 A g^−1^, and 2.0 A g^−1^; (**c**) GCD at cycle 2, 1000, 2000, and 3000; (**d**) capacitance retention and Coulombic efficiency (GCD cycles) for the SSC 1.0 M Na_2_SO_4_.

A symmetric supercapacitor was assembled using an acetate membrane soaked in 1.0 M Na_2_SO_4_ as the electrolyte (Figure 1a). Figure S4 and Figure 8a show the SSC CV curves with a potential window from ±0.4 V to ±1.5 V and scan rates from 25 mV s^−1^ to 200 mV s^−1^. The specific capacitance values are shown in Table S3. The specific capacitance for ±1.2 V increased from 16 F g^−1^ (200 mV s^−1^) to 33 F g^−1^ (25 mV s^−1^), with the highest value being 39 F g^−1^ for ±1.5 V (25 mV s^−1^). The behavior is similar to that observed for the three-electrode electrochemical cell, with an increase in current in all potential windows tested as the scan rate increases; however, the specific capacitance did not increase with an increase in scan rate. The observed drop in specific capacitance in the SC, with respect to the value measured in the three-electrode electrochemical cell, is consistent with that obtained by other authors; for example, for NiMn_2_O_4_, Sankar et al. found a drop from 202 F g^−1^ (three-electrode cell) to 50 F g^−1^ at 1 mV s^−1^ (asymmetric supercapacitor) [48].

Galvanostatic charge–discharge tests were carried out at different current densities, from 0.5 A g^−1^ to 2.0 A g^−1^, in the potential window of 0.0–1.2V (Figure 8b,c). A specific capacitance of 17 F g^−1^ was observed at a current density of 0.5 A g^−1^, yielding an energy density of 3.4 Wh kg^−1^ and a power density of 306 W kg^−1^, which positions this SSC as a supercapacitor in the Ragone plot. The specific capacitance values obtained from initial GCD cycles are similar and even higher than those found in the literature [21]. As the current density increases, the GCD curves show a more symmetric and triangular shape indicating better Coulombic efficiency, but the specific capacitance decreases due to the fast discharge of the supercapacitor. The electrochemical stability of the SSC was studied using 3000 GCD cycles at a current density of 1.0 A g^−1^ (Figure 8c). The SSC experienced a drop in specific capacitance as the number of GCD cycles increased (Figure 8c,d). Figure 8d shows the evolution of specific capacitance retention and Coulombic efficiency with the number of cycles. On the other hand, there was a significant loss of electrode material (Figures S3 and S9b) at the end of the 300 CV cycles as a consequence of the solubilization of Mn and Zn, as mentioned above.

#### 3.3.2. PVP-Ionic Liquid and PVP-LiClO_4_ as Electrolytes 

Two symmetric supercapacitors were assembled using PVP-ionic liquid or PVP-LiCLO_4_ as electrolytes following the arrangement shown in Figure 1b. The SSCs’ electrochemical tests were carried out with a two-electrode configuration.

##### PVP-Ionic Liquid Electrolyte

**Figure 9:**
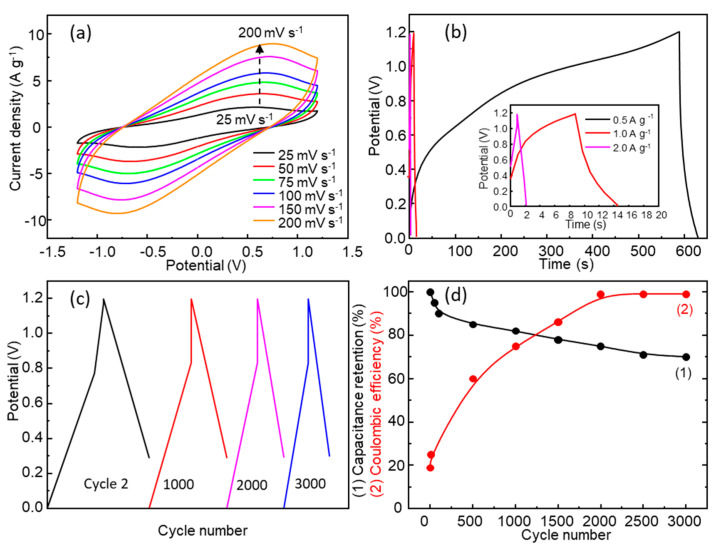
SCC with PVP-Ionic liquid: (**a**) CV cycles with a ±1.2 V potential window at scan rates from 25 to 200 mV s^−1^; (**b**) GCD at current densities of 0.5 A g^−1^, 1.0 A g^−1^, 2.0 A g^−1^; (**c**) GCD cycles 2, 1000, 2000, and 3000; (**d**) capacitance retention and Coulombic efficiency (GCD cycles).

Figure 9a and Figure S5 show the CV curves of the PVP-ionic liquid SSCs with potential windows from ±0.4 V to ±1.5 V and scan rates from 25 mV s^−1^ to 200 mV s^−1^. For the two lowest potential windows of ±0.4 V and ±0.8 V, curves were obtained that were nearly rectangular and possessed a specific capacitance of 15 F g^−1^ and 23 F g^−1^, at 25 mV s^−1^, respectively. An increase in pseudocapacitive behavior was observed for ±1.2 V (40 F g^−1^ at 25 mV/s) and ±1.5 V (37 F g^−1^ at 25 mV s^−1^), as shown in Table S4. The increase in the scan rate decreases the charge stored in the device due to the limitation of the diffusion processes, making the interaction between the electrolyte and the electrode material less effective. Figure 9b shows the electrochemical behavior of the device during GCD at different current densities and a potential window of 0–1.2 V. The highest specific capacitance of 15 F g^−1^ was obtained at 0.5 A g^−1^. At lower current densities, the charge–discharge times are longer, and the GCD profile is characteristic of a supercapacitor with faradaic contribution. As the current density increases, the GCD curve adopts a more triangular profile characteristic of EDLC (Figure 9b). Through GCD, the highest energy density of 3 Wh kg^−1^ is obtained at the current density of 0.5 A g^−1^, while the highest power density of 1080 W kg^−1^ is obtained for 2.0 A g^−1^ (Table S4). As shown in Figure 9d,e, the SSC shows good stability, maintaining a retention of 70% of its initial capacitance after 3000 GCD cycles at a current density of 1.0 A g^−1^, and the Coulombic efficiency (Figure 6d) is higher than that obtained for SSC with 1.0 M Na_2_SO_4_ (Figure 5f).

**Figure 10:**
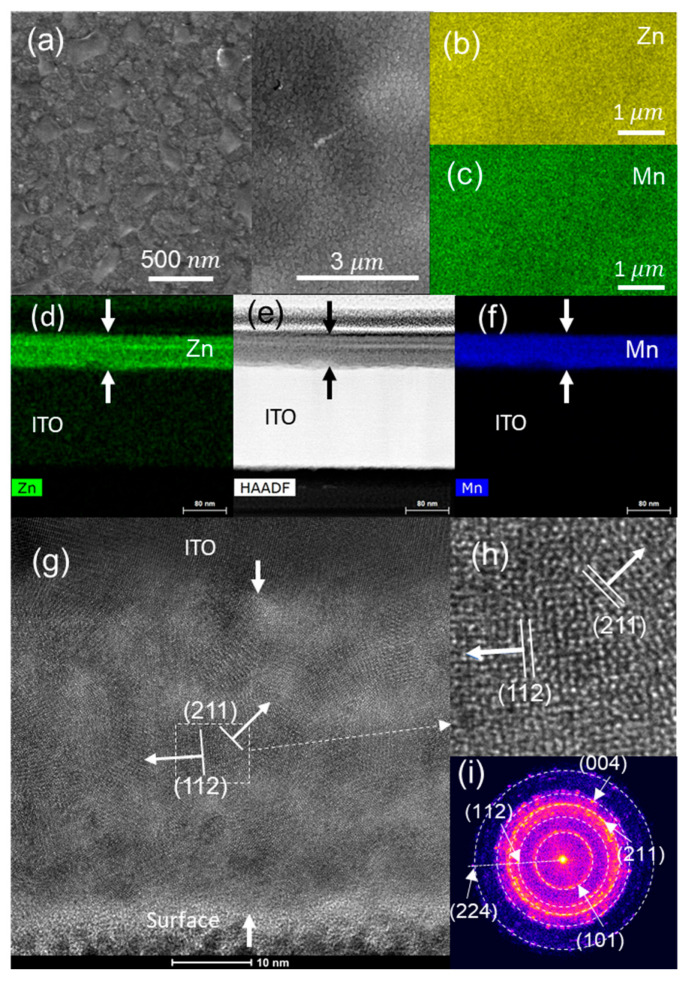
SCC with PVP-ionic liquid as electrolyte after 300 CV cycles: (**a**) SEM; (**b**) Zn and (**c**) Mn EDS images of the surface of the electrode; (**d**) Zn and (**f**) Mn EDS images; (**e**) HAADF images of the electrode cross-section; (**g**) HRTEM image of the cross-section; (**h**) magnification of the marked zone; (**i**) FFT of the film cross-section.

After 300 CV cycles, the electrodes were recovered and washed with ethanol to remove the electrolyte on the electrode surface. The SEM image (Figure 10a) shows a more textured surface than the original electrode surface (Figure 3a), with spray droplet marks still visible after 300 CV cycles. No loss of electrode material in the form of voids is observed (Figure 10a), which may be due to a lower solubility of Zn and Mn in the PVP-ionic liquid electrolyte than in the aqueous Na_2_SO_4_ electrolyte, favoring the reversibility of the reactions described in Equations (7) and (8). The EDS mappings of Mn and Zn (Figure 10b–f) show that both elements are present and homogenously distributed on the surface and in the cross-section. 

**Figure 11:**
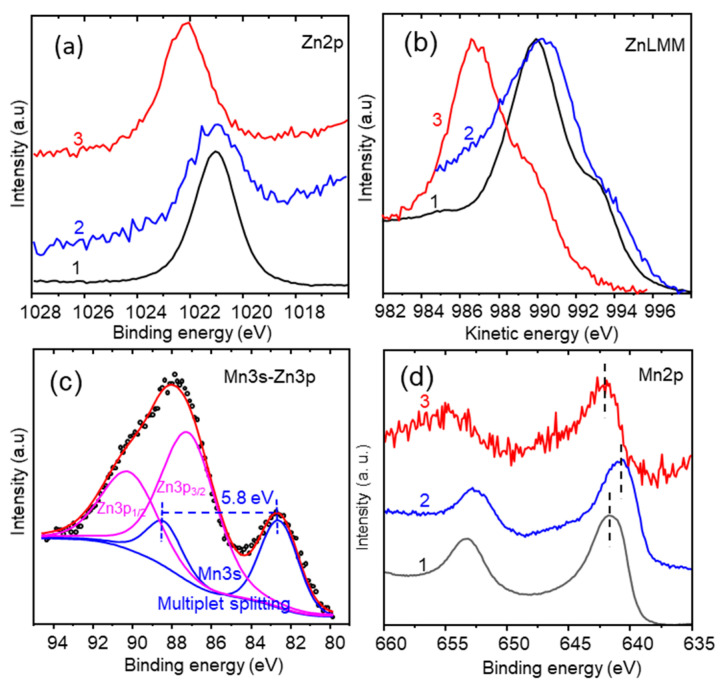
XPS of electrode of SCC PVP-Ionic Liquid: (**a**) Zn2p, (**b**) ZnLMM, (**c**) M3s Zn3p, (**d**) Mn2p. Electrodes: (1) as-deposited, (2) after 300 CV cycles finishing under reducing conditions, (3) after 300 cycles of CV finishing under oxidizing condition.

Figure 11a–d show the XPS spectra corresponding to Mn2p, Mn3s, Zn 2p_3/2_, and O1s. Both the Zn2p (Figure 11a) and Mn3s regions (Figure 11c) show a superficial loss of Zn, but this Zn loss is less than in the case of the Na_2_SO_4_ electrolyte. When the electrode was exposed to cathodic conditions at the end of the last 300th cycle of CV, the Mn2p_3/2_ and Mn3s XPS peaks (Figure 11c,d) showed a shift toward lower binding energies of 640.8 eV and 82.6 eV (641.4 eV and 83.5 eV, respectively, for the as-deposited electrode), respectively, indicating the presence of Mn^2+^ [16,65,66]. This is corroborated by the deconvolution of the Mn3s-Zn3p region (Figure 11c) with $\Delta E_{Mn3s}$ = 5.8 eV for Mn3s [42,65]. FWHMs of 2.2 eV for Mn3s and 3.3 eV for Zn3p peaks were used for the deconvolution (Figure 11c). However, when the opposite electrode was analyzed using XPS at the end of the 300th CV cycle, under more oxidizing conditions, the Mn2p peak showed a shift toward higher binding energies (642.1 eV), indicating Mn^4+^ (Figure 11d).

**Figure 12:**
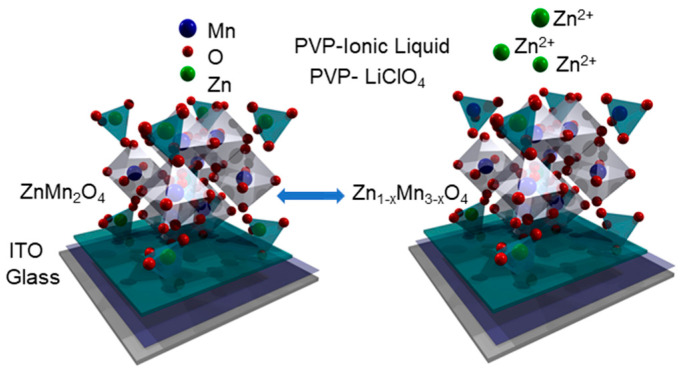
Scheme of the transformation from ZnMnO_4_ to Zn_1-x_Mn_3-x_O_4_.

These XPS results are indicative of the reversibility of the redox processes occurring on both electrodes, which can deliver a more stable capacity [67]. These valence changes at the surface are compatible with the maintenance of the tetragonal structure of the spinel, t-[Zn^2+^, Mn^2+^]o-[Mn^3+^, Mn^4+^, Zn^2+^]_2_[O]_4_ [24]. On the other hand, the ionic liquid is composed of imidazolium and the tetrafluoroborate anion (BF_4_^−^). Furthermore, the XPS Zn2p (Figure 11a) and the Auger region ZnLMM (Figure 11b) show a displacement in the binding energy to 1022 eV for Zn2p_3/2_ and 986.7 eV (kinetic energy) for ZnLMM, values identified as corresponding to ZnF_2_ [68]. However, in this case, these values would correspond to Zn^2+^[BF_4_^−^], which is proof of the chemical interaction between the electrode material and the electrolyte and is responsible for the partial solubilization of Zn in the electrode–quasi-solid-electrolyte interface. According to FFT HRTEM of the cross-section (Figure 10g–i), crystalline planes were identified that are compatible with a transformation from hetaerolite ZnMn_2_O_4_ (PDF 01-071-2499) to Mn-rich tetragonal phases such as Zn_0.75_Mn_2.25_O_4_ (PDF 04-016-9607), with the stoichiometry changing across the cross-section (Figure S6). Figure S6b shows the evolution of the Mn/Zn atomic ratio in the cross-section. Figure 12 shows a scheme illustrating this transformation from ZnMn_2_O_4_ to the Mn-rich phase Zn_1−x_Mn_3−x_O_4_. 

##### PVP-LiClO_4_ Electrolyte

**Figure 13:**
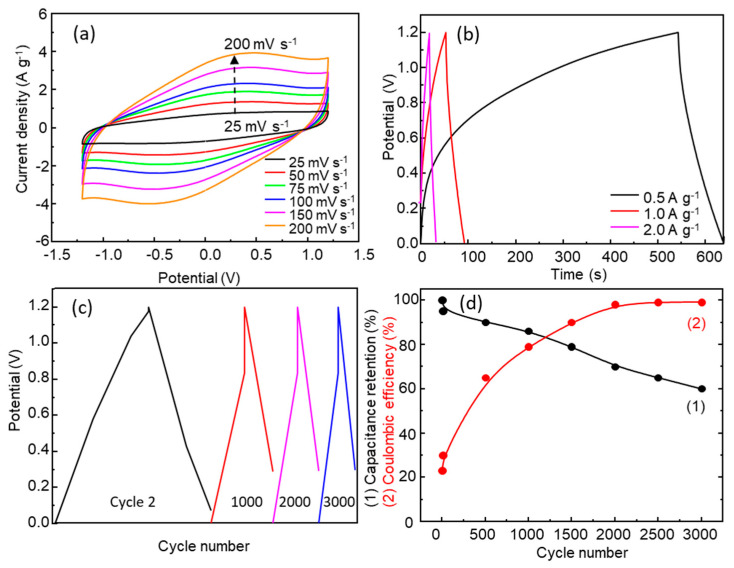
SCC with PVP-LiClO_4_: (**a**) CV cycles with a ±1.2 V potential window at scan rates from 25 to 200 mV s^−1^; (**b**) GCD at current densities of 0.5 A g^−1^, 1.0 A g^−1^, 2.0 A g^−1^; (**c**) GCD cycles 2, 1000, 2000, and 3000; (**d**) capacitance retention and Coulombic efficiency (GCD cycles).

Figure S7 and Figure 13a show the CV curves with potential windows from ±0.4 V to ±1.5 V and scan rates from 25 mV s^−1^ to 200 mV s^−1^ (Table S5). The highest CV specific capacitance value was 22 F g^−1^ for a scan rate of 25 mV s^−1^ and a potential window of ±1.2 V. Figure 13b shows the results of the GCD tests at constant current densities of 0.5 A g^−1^, 1.0 A g^−1^, and 2.0 A g^−1^ using a potential window of 0–1.2 V. 

The SCC with this electrolyte shows the longest charge and discharge time of the three electrolytes. The discharge time and the specific capacitance were 94 s and 39 F g^−1^, respectively, for an extracted current density of 0.5 A g^−1^ (Figure 13b). At higher current density, the SSC shows a reduction in discharge time and capacitance (Table S5) due to the inability of the electrolyte to sustain a high charge transfer rate. The SSC using PVP-LiClO_4_ retained 60% of its initial specific capacity after 3000 GCD cycles (Figure 13d). A factor that can influence the loss of capacitance retention may be the fact that when the residual amount of ethanol used in the PVP solution that remains in the solid electrolyte is lost, the ionic conductivity of the PVP-LiClO_4_ solid electrolyte decreases [33].

**Figure 14:**
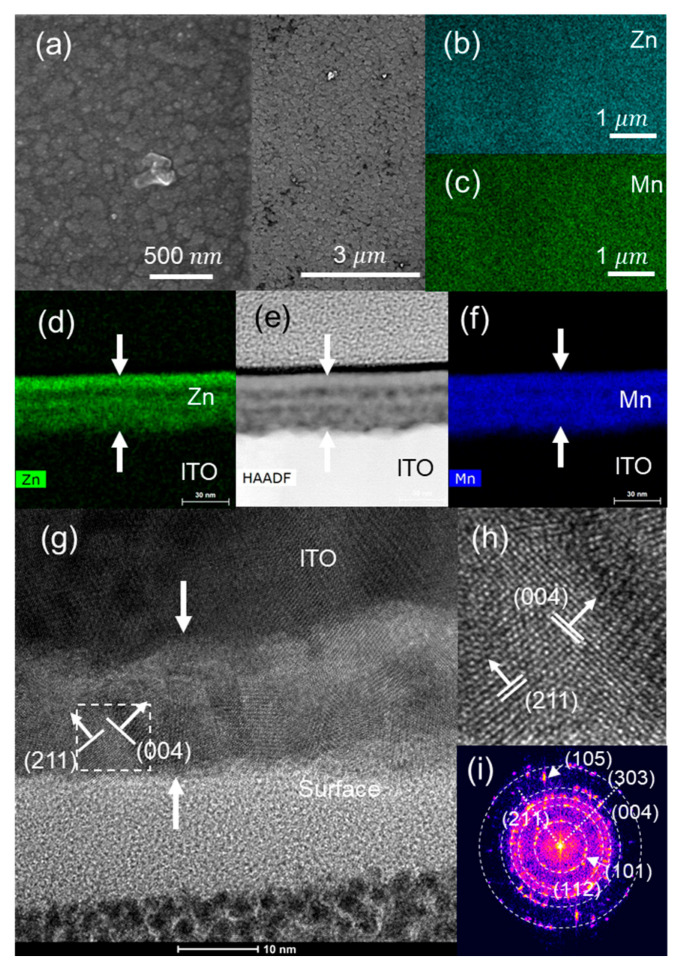
SCC with PVP-LiClO_4_ as electrolyte after 300 CV cycles: (**a**) SEM; (**b**) Zn and (**c**) Mn EDS images of the surface of the electrode; (**d**) Zn and (**f**) Mn EDS images; (**e**) HAADF images of the electrode cross-section; (**g**) HRTEM image of the cross-section; (**h**) magnification of the marked zone; (**i**) FFT of the film cross-section.

After 300 CV cycles, the electrodes of the SSC were recovered and washed with ethanol to remove the electrolyte on the electrode surface. SEM images (Figure 14a) of the electrode after 300 CV cycles with anodic conditions at the end of the last CV cycle show a more textured surface than the original electrode surface (Figure 3a). SEM-EDS images (Figure 14b,c) show a homogeneous distribution of Mn and Zn on the electrode surface, the same as the TEM-EDS image of the cross-section (Figure 14d,f). 

**Figure 15:**
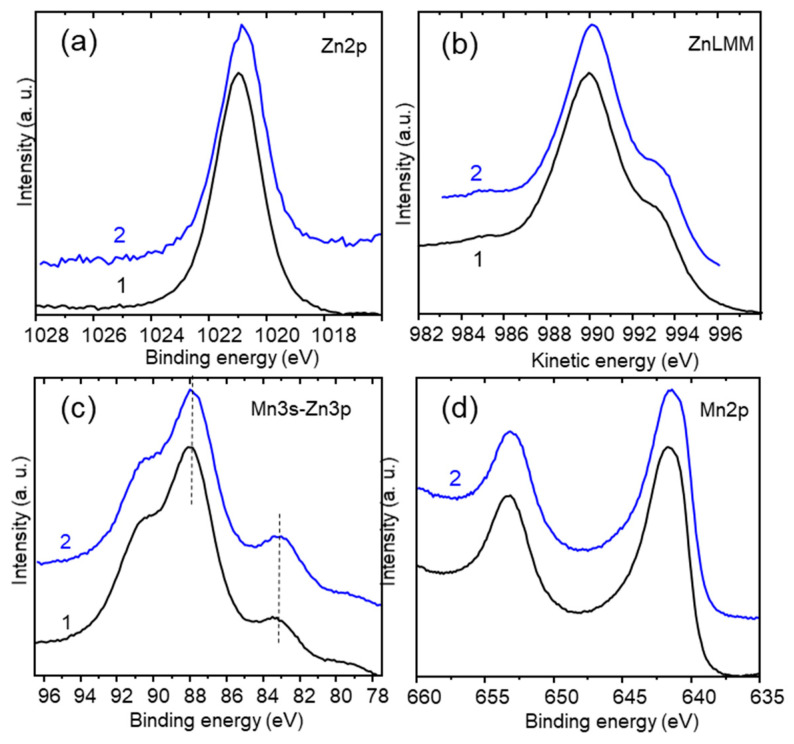
XPS of electrode of SCC PVP-LiClO_4_: (**a**) Zn2p, (**b**) ZnLMM, (**c**) M3s Zn3p, (**d**) Mn2p. Electrodes: (1) as-deposited, (2) after 300 CV cycles finishing under reducing conditions.

The XPS Zn2p (Figure 15a) and the ZnLMM spectra (Figure 15b) are similar to those of the corresponding original spinel surface. The Mn2p and M3s-Zn3p peaks are also similar to those of the ZnMn_2_O_4_ spinel. After the 300th CV cycle, the Mn2p_3/2_ region of the electrode finished in cathodic conditions shows a very slight contribution to lower binding energy, 0.2 eV, corresponding to the presence of reduced Mn valences (Figure 15d). The XPS spectra of the Zn2p, ZnLMM, and Zn3p-Mn3s regions of the electrode after 300 cycles of CV are very similar to those obtained for the electrode before CV. If we compare this with what occurred with the other two electrolytes, the ZnMn_2_O_4_ spinel in the SCC with PVP-LiClO_4_ was shown to be more stable after 300 CV cycles. Using the fast Fourier-transform (FFT) HRTEM of the cross-section (Figure 14g,h), it is possible to identify crystalline planes compatibles with a mix of spinel ZnMn_2_O_4_ (PDF 01-071-2499) and Mn-rich tetragonal phases, such as Zn_0,75_Mn_2,25_O_4_ (PDF 04-016-9607), with a stoichiometry change across the cross-section (Figure S8). In this case, due to the fact that Zn^2+^ has a similar ionic radius (0.74 Å) to that of Li^+^ (0.76 Å), the intercalation of Li^+^ into the spinel structure could play a role in the faradaic processes [69].

**Figure 16:**
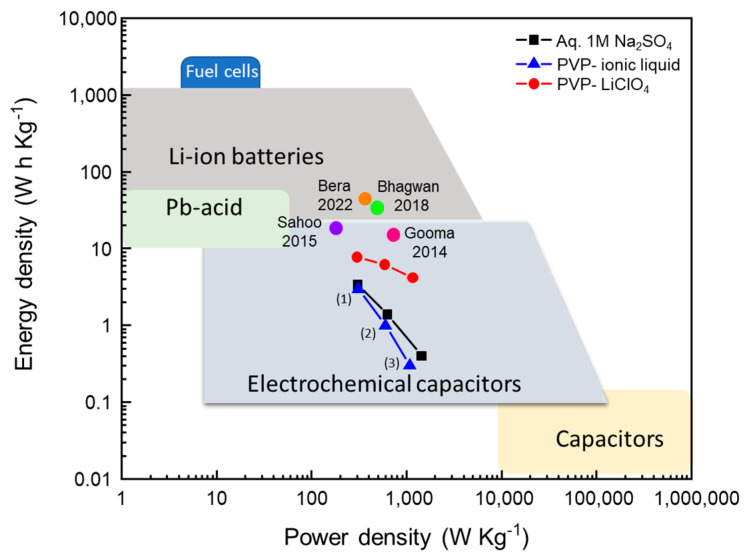
Ragone plot, points corresponding to (1) 0.5 A g^−1^, (2) 1.0 A g^−1^, (3) 2.0 A g^−1^, and values obtained by other authors [70,71,72,73].

Figure 16 shows the Ragone plot at 0.5 A g^−1^, 1.0 A g^−1^, and 2.0 A g^−1^ for the three SCC_S_. All of them showed supercapacitor behavior. 

## 4. Conclusions

ZnMn_2_O_4_ thin films were prepared using the spray pyrolysis method and used as transparent electrodes in symmetric supercapacitors. The electrical resistivity of the films limited the film thickness of ZnMn_2_O_4_. ZnMn_2_O_4_ thin films were tested in a three-electrode electrochemical cell with aqueous 1.0 M Na_2_SO_4_ as the electrolyte, showing a specific capacity of 697 F g^−1^ and a specific capacity retention of 70% after 3000 GCD cycles. The challenge remains to ensure that the specific capacitance obtained with the three-electrode electrochemical cell does not decrease when the electrode is used in the supercapacitor. Of the three electrolytes tested, the SCC with 1.0 M Na_2_SO_4_ showed the best specific capacitance, but also showed the lowest capacitance retention due to irreversible Zn loss and corrosion of the active electrode material. PVP-ionic liquid and PVP-LiClO_4_ electrolytes preserve the tetragonal spinel structure of the electrode with changes in stoichiometry to a Mn-rich Zn_1-x_M_3-x_O_4_, which can be interpreted as a semi-reversible process of Zn^2+^ deintercalation/intercalation without evidence of a loss of active electrode material along the CV cycles. In the case of the electrolyte 1.0 M Na_2_SO_4_, the loss of Zn^2+^ leads to the formation of MnO_2_ via Zn_1-x_M_3-x_O_4_. XPS analysis of the two electrodes forming the symmetric supercapacitor shows that there is a change in the Mn valence on the electrode surface, with the presence of Mn^4+^ or Mn^2+^ depending on if, at the end of the final cycle, the electrode was exposed to more oxidizing or reducing conditions. It is observed in the SCC PVP-ionic liquid that, on the electrode surface, there is an interaction between the anion [BF_4_^−^] of the ionic liquid and the Zn^2+^. The Ragone plot (Figure 16) of the three SSCs shows supercapacitor behavior; pseudocapacitance is the main contributor. The electrochemical results prove that pseudocapacitance is the major contributor to the electrode capacitance and that SCCs can therefore be considered as pseudocapacitors.

## Data Availability

The data presented in this study are available upon request from the corresponding author.

## Supplementary Materials

The following supporting information can be downloaded at https://www.mdpi.com/article/10.3390/nano13233017/s1; Figure S1: Three-electrode cyclic voltammogram curves at different scan rates: 5, 10, 25, 50, 75, 100, 150, and 200 mV s^−1^; Table S1: Values of specific capacitance obtained by different authors; Figure S2: Example of EDS spectrum of the ZnMn_2_O_4_ electrode as obtained, and atomic percentages of Mn and Zn; Figure S3: Example of EDS spectrum of the electrode after 3000 CV cycles and local values of the atomic percentage of the different chemical elements; Figure S4: Cyclic voltammetry curves of SSC assembled with 1.0 M Na_2_SO_4_. (a) ±0.4 V; (b) ±0.8 V; (c) ±1.2 V; (d) ±1.5 V with different scan rates: 25 mV s^−1^, 50 mV s^−1^, 75 mV s^−1^, 100 mV s^−1^, 150 mV s^−1^, and 200 mV s^−1^; Table S2: (a) Specific capacitance, energy, and power density calculated from cyclic voltammetry; (b) GCD measurements of the SSC assembled with 1.0 M Na_2_SO_4_ electrolyte; Figure S5: Cyclic voltammetry curves of SSC assembled with PVP-ionic liquid. (a) ±0.4 V; (b) ±0.8 V; (c) ±1.2 V; (d) ±1.5 V with different scan rates: 25 mV s^−1^, 50 mV s^−1^, 75 mV s^−1^, 100 mV s^−1^, 150 mV s^−1^, and 200 mV s^−1^; Table S3: (a) Specific capacitance, energy, and power density calculated from cyclic voltammetry and (b) GCD measurements of the SSC assembled with PVP-ionic liquid; Figure S6: (a) HAADF image EDS of the cross-section of the SCC (PVP-ionic liquid) electrode after 300 CV cycles; (b) Mn/Zn atomic ratio and (c) atomic percentages of Mn and Zn, along the line marked on the HAADF image; Figure S7: Cyclic voltammetry curves of SSC assembled with PVP-ionic liquid. (a) ±0.4 V; (b) ±0.8 V; (c) ±1.2 V; (d) ±1.5 V with different scan rates: 25 mV s^−1^, 50 mV s^−1^, 75 mV s^−1^, 100 mV s^−1^, 150 mV s^−1^, and 200 mV s^−1^; Table S4: (a) Specific capacitance, energy, and power density calculated from cyclic voltammetry; (b) galvanostatic charge–discharge measurements of the SSC assembled with PVP-LiClO_4_; Table S5: (a) Specific capacitance, energy and power density calculated from cyclic voltammetry; (b) galvanostatic charge-discharge measurements of the SSC assembled with PVP-LiClO_4_; Figure S8: (a) HAADF image EDS of the cross-section of the SCC electrode (PVP-LiClO_4_) after 300 CV cycles; (b) Mn/Zn atomic ratio; and (c) atomic percentages of Mn and Zn, along the line marked on the HAADF image; Figure S9: (a) SEM images of the surface of the electrode as obtained. SEM images of the surface of the SSC electrode after 300 CV cycles using as electrolyte: (b) 1.0 M Na_2_SO4, (c) PVP-ionic liquid, and (d) PVP-LiClO_4_ [2,45,70,74,75,76,77,78].
