# Supplementary material for: Effect of the Nature of the Electrolyte on the Behavior of Supercapacitors Based on Transparent ZnMn2O4 Thin Films
Source: Nanomaterials (Basel). 2023 Nov 24;13(23):3017. doi: 10.3390/nano13233017 (PMC10708517; doi:10.3390/nano13233017)
Supplement: Supplementary file 1 [file nanomaterials-13-03017-s001.zip › nanomaterials-2714841-supplementary.pdf]

Supplementary Information

Effect of the nature of the electrolyte on the behaviour of supercapacitors based on transparent $ZnMn_2O_4$ thin films

Juan José Peinado-Pérez[a], María Cruz Lopez-Escalante[b], Francisco Martín[b*]

[a]*Departament of Applied Physics I. Faculty of Sciences, University of Málaga, Campus de Teatinos, E-29071. Málaga (Spain).*
[b]*Departament of Chemical Engineering, Faculty of Sciences, University of Málaga, Campus de Teatinos, E-29071 Málaga, Spain. (* corresponding author marjim@uma.es)*

1. Optical properties

Sample	Visible transmittance (from 380 to 770 nm) D65	Solar transmittance (from 295 to 2415 nm)	Colorimetric coordinates CIE 1931	Colorimetric coordinates CIELAB (1976, 2º, D65)
**ITO**	0.82	0.79	Y=81.81, x=0.31, y=0.32	L*= 92.49, a* =1.03, b* =-3.01
**ITO/$ZnMn_2O_4$ (2 min)**	0.72	0.71	Y=71,29, x=0.33, y=0.35	L*= 87.83, a* =-1.16, b* =8,73
**ITO/$ZnMn_2O_4$ (5 min)**	0.57	0.63	Y=56.6, x=0.35, y=0.36	L*= 79.95, a* =0.71, b* =16.62
**ITO/$ZnMn_2O_4$ (15 min)**	0.30	0.48	Y=30.49, x=0,4, y=0.4	L*= 62.07, a* =5.72, b* = 30.81

Table SI 1: Visible and solar transmittance values, and colorimetric coordinates

2. Three-electrode electrochemical cell characterization of $ZnMn_2O_4$ electrodes Figure SI 1:

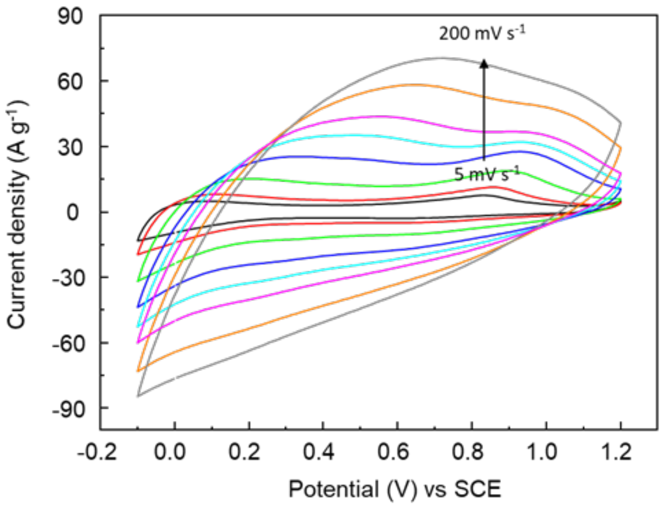

Three-electrode cyclic voltammogram curves at different scan rates: 5, 10, 25, 50, 75, 100, 150 and 200 mV s^{-1}.

Deposition method	Electrode	Electrolyte	Specific capacitance (F g^{-1})	Capacitance retention (%)	Energy density (W h kg^{-1})	Power density (W kg^{-1})	Reference
Pneumatique Spray deposition	$ZnMn_2O_4$	1M Na_2SO_4	752 (0.5A g^{-1}) 693 (5 mV s^{-1})	70% after 3000 cycles	1-8	300-1000	This study
	$ZnMn_2O_4$	2M KOH	155 (2 mV s^{-1})	*	*	*	[45]
Hydrothermal method	$ZnMn_2O_4$	1M KCl	675 (5 mV s^{-1})	61.7 % after 1000 Cycles	*	*	[74]
	$ZnMn_2O_4$	1M Na_2SO_4	87 (1 mV s^{-1})	60 % after 1000 Cycles	*	*	[75]
	$ZnMn_2O_4$	2 M KOH	54 (20 mV s^{-1})	*	*	*	[76]
	$ZnMn_2O_4$	2 M KOH	776 (5 mV s^{-1})	91 % after 5000 cycles	*	*	[77]
Combustion method	Carbon-$ZnMn_2O_4$	Na_2SO_4	150 (2 mV s^{-1})	60 % after 10000 cycles	*	*	[2]
	$ZnMn_2O_4$	2 M KOH	160 (3 mV s^{-1})	100% after 500 cycles	18	185	[70]
Ultrasonication	$ZnMn_2O_4$-Mn_2O_3	2 M KOH	380 (0.5 A g^{-1})	92 % after 2000 cycles	*	*	[78]
* Non data available							

Table SI 2: Values of specific capacitance obtained by different authors

References

2. Abdollahifar, M.; Huang, S.S.;Y.H. Lin, Y.H.; Li, Y.C.; Shih, B.Y.; Sheu, H.S.; Liao, Y.F.; Wu, N.I. High-performance carbon-coated ZnMn2O4 nanocrystallite supercapacitors with tailored microstructures enabled by a novel solution combustion method, J. Power Sources. 2018, 378, 90-97. https://doi.org/10.1016/j.jpowsour.2017.12.022.

45. Guo, N.; Wei, X.; Deng, X.L.; Xu, X.J. Synthesis and Property of Spinel Porous $ZnMn_2O_4$ microspheres, Appl. Surf. Sci. 2015, 356, 1127-1134. https://doi.org/10.1016/j.apsusc.2015.08.185

70. Sahoo, A.; Sharma. Y. Synthesis and characterization of nanostructured ternary zinc manganese oxide as novel supercapacitor material. Mater. Chem. Phys, 2015, 149–150, 721-727. https://doi.org/ 10.1016/j.matchemphys.2014.11.032.

74. Senthilkumar, N.; Venkatachalam, V.; Kandiban, M.; Vigneshwaran, P.; Jayavel, R.; Potheher, I.V. Studies on Electrochemical Properties of Hetarolite ($ZnMn_2O_4$) Nanostructure for Supercapacitor Application. Physica E: Low-Dimens. Syst. Nanostruct., 2019, 106, 121-126. https://doi.org/10.1016/j.physe.2018.10.027.

75. Aruchamy, K.; Nagaraj, R.; Manohara , H.M.; Nidhi , M.R.; Mondal , D.; Ghosh , D.; Nataraj. K. One-step green route synthesis of spinel $ZnMn_2O_4$ nanoparticles decorated on MWCNTs as a novel electrode material for supercapacitor. Mater. Sci. Eng. B, 2020, 252. https://doi.org/10.1016/j.mseb.2019.114481.

76. Wei, X.Q.; Wang, Y.L.; Guo, N.; Deng, X.; Xu. X.J. Effect of growing temperature on structure and electrochemical performance of $ZnMn_2O_4$ nanospheres. Ionics, 2017, 23, 2443–2448. https://doi.org/ 10.1007/s11581-017-2082-1

77. Mani, M.P; Venkatachalam, V.; Thamizharasan, K.; Jothibas. M.Evaluation of Cubic-Like Advanced ZnMn2O4 Electrode for High-Performance Supercapacitor Applications. J. Electron. Mater., 2021, 50, 4381–4387. https://doi.org/10.1007/s11664-021-08962-0

78.Sannasi, V.; Subbian, K. A facile synthesis of ZnMn2O4/Mn2O3 composite nanostructures for supercapacitor applications. Ceram. Int., 2021, 47, 12300-12309. https://doi.org/10.1016/j.ceramint.2021.01.081

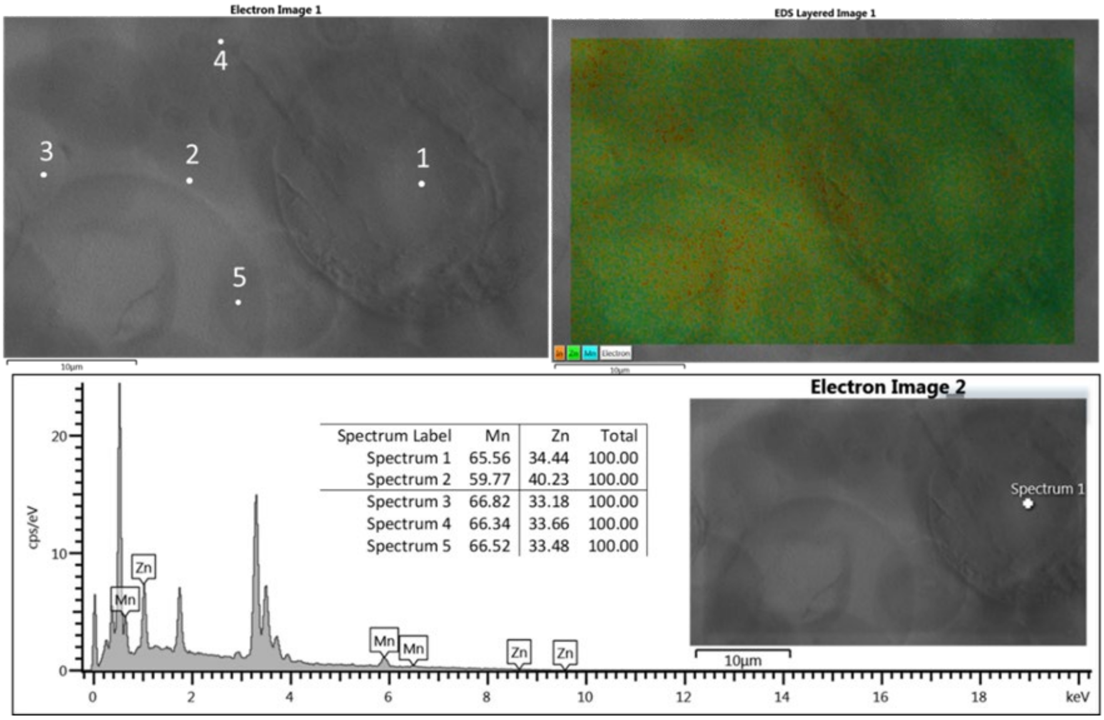

Spectrum Label	Mn	Zn	Total
Spectrum 1	65.56	34.44	100.00
Spectrum 2	59.77	40.23	100.00
Spectrum 3	66.82	33.18	100.00
Spectrum 4	66.34	33.66	100.00
Spectrum 5	66.52	33.48	100.00

Figure SI 2: Example of EDS spectrum of the $ZnMn_2O_4$ electrode as obtained, and atomic percentages of Mn and Zn.

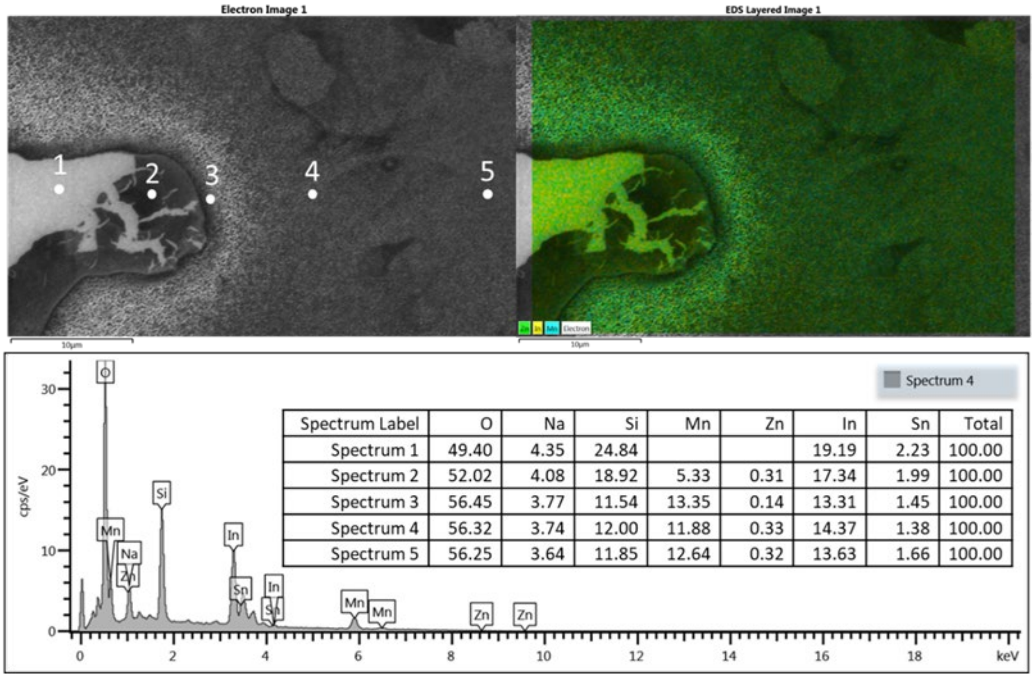

Spectrum Label	O	Na	Si	Mn	Zn	In	Sn	Total
Spectrum 1	49.40	4.35	24.84			19.19	2.23	100.00
Spectrum 2	52.02	4.08	18.92	5.33	0.31	17.34	1.99	100.00
Spectrum 3	56.45	3.77	11.54	13.35	0.14	13.31	1.45	100.00
Spectrum 4	56.32	3.74	12.00	11.88	0.33	14.37	1.38	100.00
Spectrum 5	56.25	3.64	11.85	12.64	0.32	13.63	1.66	100.00

Figure SI 3: Example of EDS spectrum of the $ZnMn_2O_4$ electrode after 3000 CV cycles and local values of the atomic percentage of the different chemical elements.

3. SCC, 1 M Na_2SO_4 electrolyte

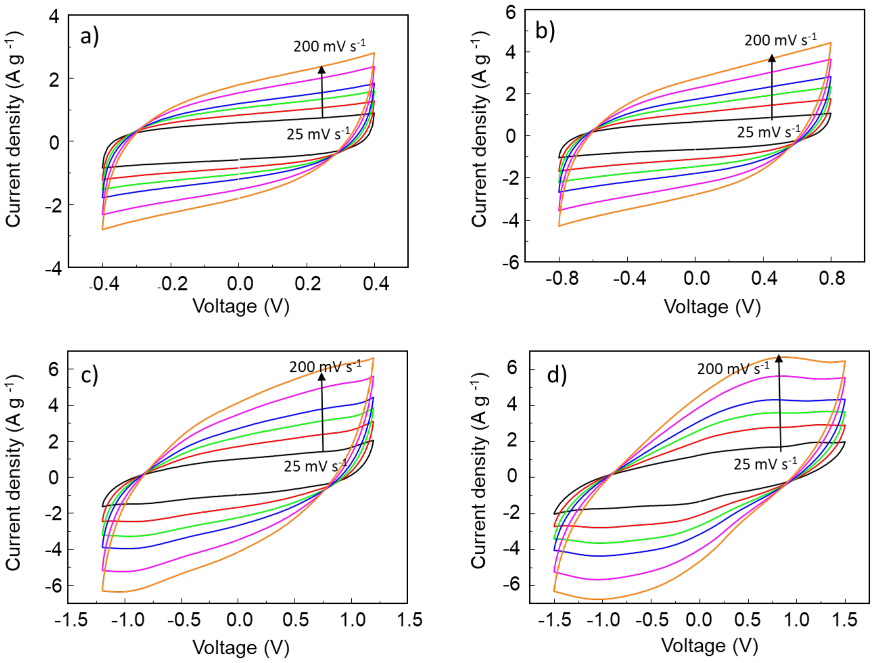

Figure SI 4: Cyclic voltammetry curves of SSC assembled with 1 M Na_2SO_4. (a) ±0.4 V; (b) ±0.8 V; (c) ±1.2 V; d) ±1.5 V with different scan rates: 25 mV s^{-1}, 50 mV s^{-1}, 75 mV s^{-1}, 100 mV s^{-1}, 150 mV s^{-1} and 200 mV s^{-1}.

(a)

Specific capacitance (Fg^{-1})	Scan rate (mVs^{-1})	Potential window (V)			
		±0.4	±0.8	±1.2	±1.5
1M Na_2SO_4	25	21	23	33	39
	50	15	19	28	30
	75	12	17	24	26
	100	10	15	21	23
	150	9	13	18	20
	200	8	11	16	17

(b)

Electrolyte	Current Density (A g^{-1})	Time discharge (s)	Specific Capacitance (F g^{-1})	Energy density (Wh kg^{-1})	Power density (W kg^{-1})
1M Na_2SO_4	0.5	40	17	3.4	306
	1.0	8	7	1.4	630
	2.0	1	2	0.4	1440

Table SI 3. (a) Specific capacitance, energy and power density calculated from cyclic voltammetry; (b) GCD measurements of the SSC assembled with 1.0 M Na_2SO_4 electrolyte

4. SCC, PVP-Ionic Liquid electrolyte

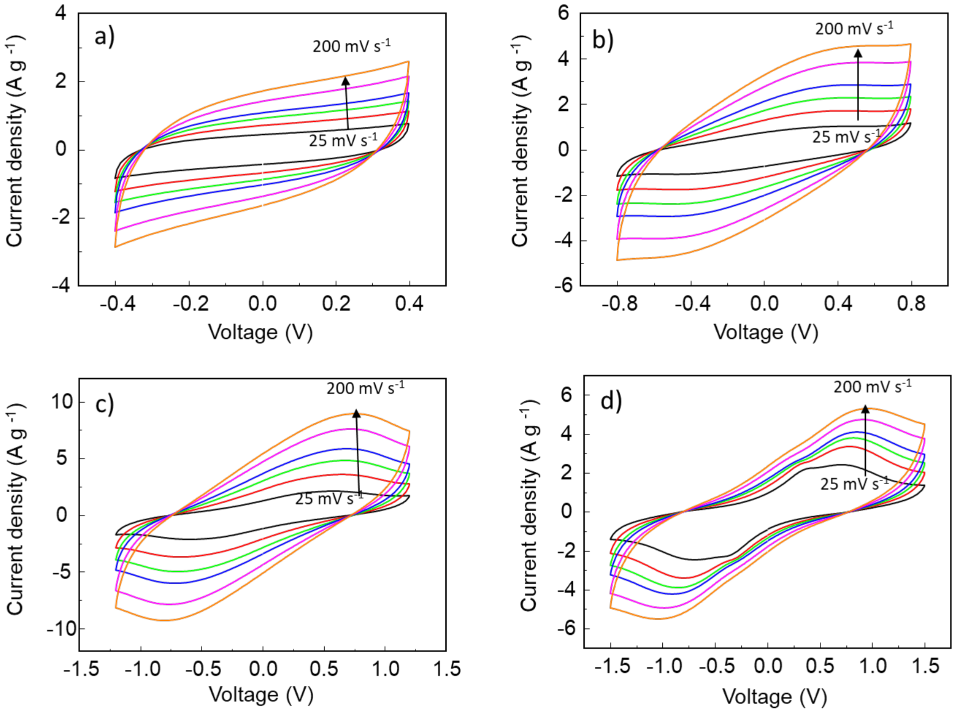

Figure SI 5. Cyclic voltammetry curves of SSC assembled with PVP-Ionic Liquid. (a) ±0.4 V; (b) ±0.8 V; (c) ±1.2 V; (d) ±1.5 V with different scan rates: 25 mV s^{-1}, 50 mV s^{-1}, 75 mV s^{-1}, 100 mV s^{-1}, 150 mV s^{-1} and 200 mV s^{-1}.

(a)

Specific capacitance (Fg^{-1})	Scan rate (mVs^{-1})	Potential window (V)			
		±0.4	±0.8	±1.2	±1.5
PVP-Ionic Liquid	25	15	23	40	37
	50	11	20	35	29
	75	10	18	31	20
	100	9	16	28	16
	150	8	14	24	13
	200	7	13	21	11

(b)

Electrolyte	Current Density (A g^{-1})	Time discharge (s)	Specific Capacitance (F g^{-1})	Energy density (Wh kg^{-1})	Power density (W kg^{-1})
PVP-Ionic Liquid	0.5	35	15	3.0	309
	1.0	6	5	1.0	600
	2.0	2	3	0.6	1080

Table SI 4. (a) Specific capacitance, energy and power density calculated from cyclic voltammetry, (b) GCD measurements, of the SSC assembled with PVP-Ionic Liquid.

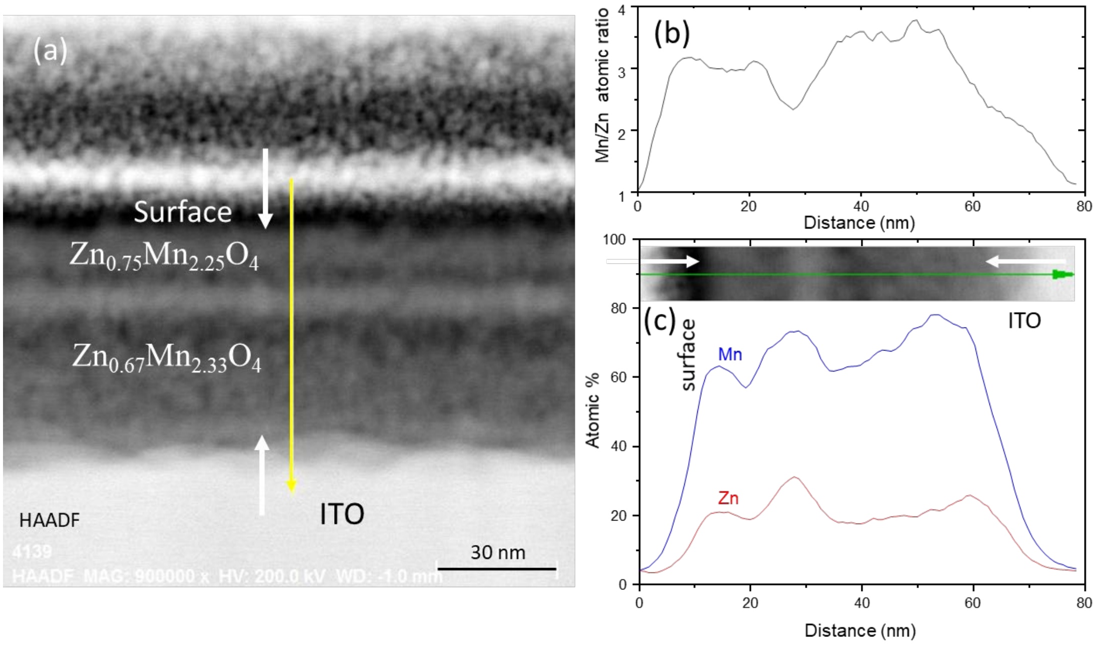

Figure SI 6: (a) HAADF image EDS of the cross-section of the SCC (PVP-Ionic Liquid) electrode after 300 CV cycles; (b) Mn/Zn atomic ratio and (c) atomic percentages of Mn and Zn, along the line marked on the HAADF image.

5. SCC, PVP-LiClO$_4$ electrolyte

Figure SI 7. Cyclic voltammetry curves of SSC assembled with PVP-Ionic Liquid. (a) ±0.4 V; (b) ±0.8 V; (c) ±1.2 V; (d)

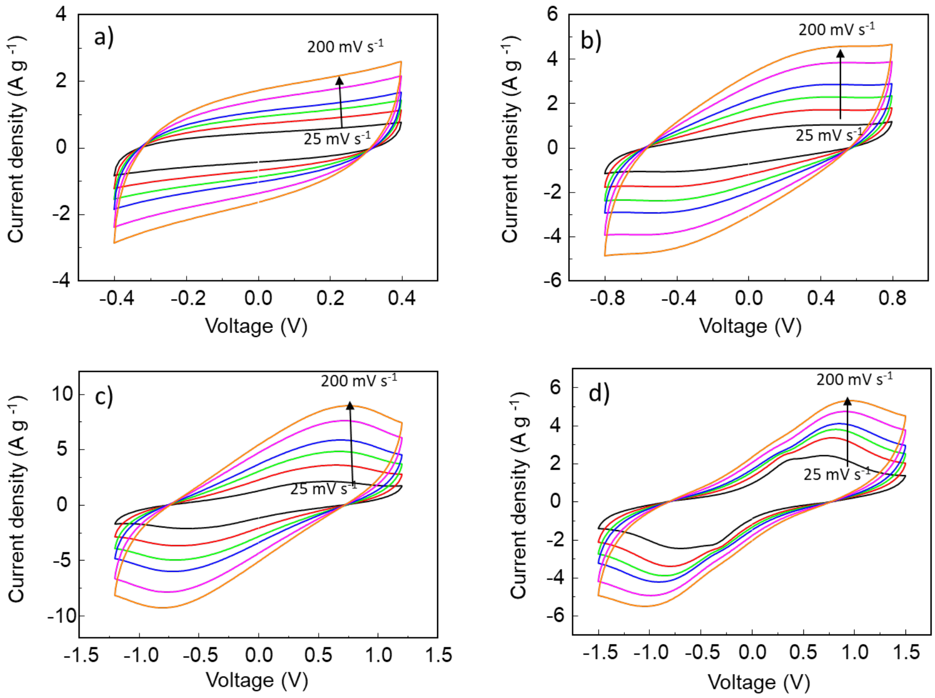

±1.5 V with different scan rates: 25 mV s^{-1}, 50 mV s^{-1}, 75 mV s^{-1}, 100 mV s^{-1}, 150 mV s^{-1} and 200 mV s^{-1}.

(a)					
Specific capacitance (F g^{-1})	Scan rate (mVs^{-1})	Potential window (V)			
		±0.4	±0.8	±1.2	±1.5
$PVP\text{-}LiClO_4$	25	13	17	22	19
	50	12	16	19	16
	75	11	15	17	14
	100	10	14	15	12
	150	10	14	14	11
	200	9	13	13	10

(b)

Electrolyte	Current Density (A g^{-1})	Time discharge (s)	Specific Capacitance (F g^{-1})	Energy density (Wh kg^{-1})	Power density (W kg^{-1})
$PVP\text{-}LiClO_4$	0.5	94	39	7.8	299
	1.0	38	31	6.2	587
	2.0	13	21	4.2	1163

Table SI 5. (a) Specific capacitance, energy and power density calculated from cyclic voltammetry; (b) galvanostatic charge-discharge measurements of the SSC assembled with $PVP\text{-}LiClO_4$.

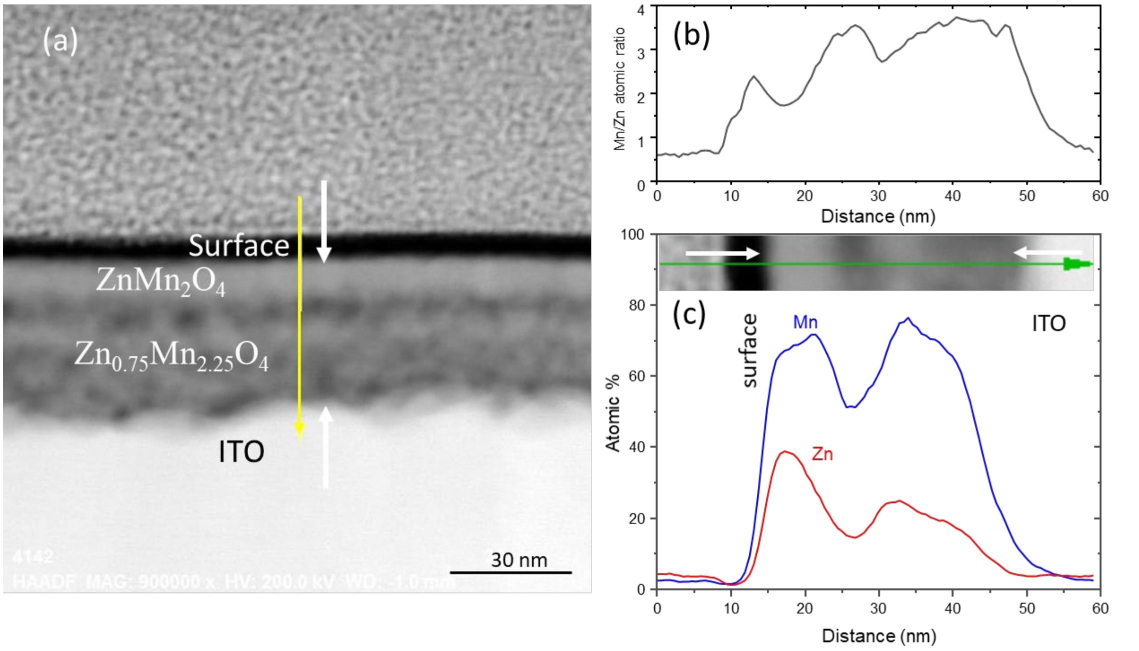

Figure S8: (a) HAADF image EDS of the cross-section of the SCC electrode ($PVP\text{-}LiClO_4$) after 300 CV cycles; (b) Mn/Zn atomic ratio and (c) atomic percentages of Mn and Zn, along the line marked on the HAADF image

6. SEM images of the SSCs electrodes after CV cycling

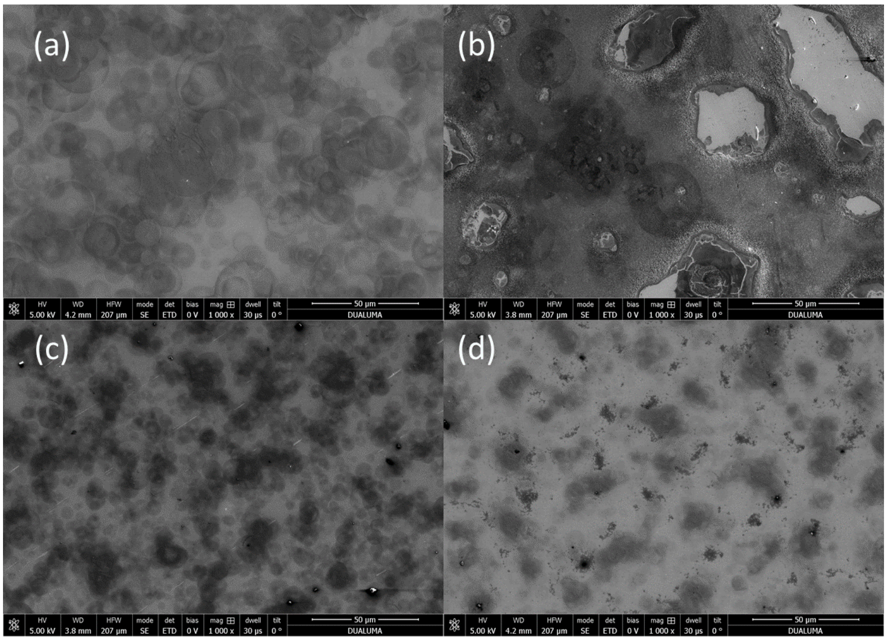

Figure SI 9: (a) SEM images of the surface of the electrode as obtained. SEM images of the surface of the SSC electrode after 300 CV cycles using as electrolyte: (b) 1.0 M Na_2SO_4, (c) PVP-Ionic Liquid, (d) PVP-$LiClO_4$.
